# Cytotoxicity of Amino‐BODIPY Modulated via Conjugation with 2‐Phenyl‐3‐Hydroxy‐4(1*H*)‐Quinolinones

**DOI:** 10.1002/open.202100025

**Published:** 2021-08-23

**Authors:** Martin Porubský, Kristýna Vychodilová, David Milićević, Miloš Buděšinský, Jarmila Stanková, Petr Džubák, Marián Hajdúch, Jan Hlaváč

**Affiliations:** ^1^ Department of Organic Chemistry Faculty of Science Palacký University Tř. 17. Listopadu 12 771 46 Olomouc Czech Republic; ^2^ Institute of Molecular and Translational Medicine Faculty of Medicine and Dentistry Palacký University Hněvotínská 5 779 00 Olomouc Czech Republic; ^3^ Institute of Organic Chemistry and Biochemistry of the Czech Academy of Sciences Flemingovo nám. 542/2 160 00 Prague Czech Republic

**Keywords:** amino-BODIPY dyes, cytotoxic activity, disulfide linkers, glutathione, hydroxyquinolinones

## Abstract

The combination of cytotoxic amino‐BODIPY dye and 2‐phenyl‐3‐hydroxy‐4(*1H*)‐quinolinone (3‐HQ) derivatives into one molecule gave rise to selective activity against lymphoblastic or myeloid leukemia and the simultaneous disappearance of the cytotoxicity against normal cells. Both species′ conjugation can be realized via a disulfide linker cleavable in the presence of glutathione characteristic for cancer cells. The cleavage liberating the free amino‐BODIPY dye and 3‐HQ derivative can be monitored by ratiometric fluorescence or by the OFF‐ON effect of the amino‐BODIPY dye. A similar cytotoxic activity is observed when the amino‐BODIPY dye and 3‐HQ derivative are connected through a non‐cleavable maleimide linker. The work reports the synthesis of several conjugates, the study of their cleavage inside cells, and cytotoxic screening.

## Graphical abstract

**Amino‐BODIPY conjugates** with various 2‐phenyl‐3‐hydroxyquinolinones (3‐HQs) connected via cleavable disulfide linker and non‐cleavable maleimide linker were prepared and tested on cytotoxic activity and fluorescent ratiometric “OFF‐ON” monitoring of cleavage inside the cells was demonstrated. Disulfide conjugates exhibited improved cytotoxicity compared to the free 3‐HQs whereas non‐cleavable maleimide conjugates stayed inactive proving that disulfide linker is responsible for cleavage inside the cells and therefore resulting in cytotoxicity of disulfide Amino‐BODIPY‐3HQ conjugates.

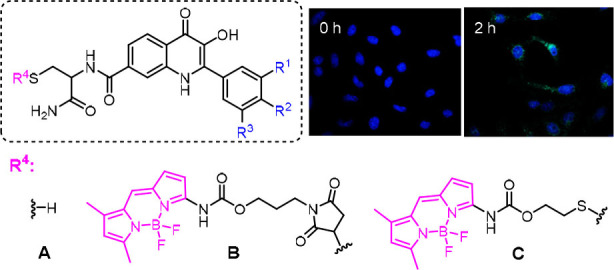

## 1 Introduction

Fluorescent dyes conjugated with other molecules belong to essential bioimaging tools for several decades.[^1^, ^2^, ^3^, ^4^, ^5^, ^6^, ^7^, ^8^, ^9^, ^10^] Their role in visualizing the appropriate process and detecting or determining the desired analyte is irreplaceable in contemporary chemical biology. As they have been extensively used in in vitro as well as in vivo assays, their toxicity should not affect the biological processes in the living system.

One of the most used dyes in fluorescent labeling and monitoring is the boron‐dipyrromethene dye, frequently called BODIPY. Its derivatives have been used several times for detection of pH,[^11^, ^12^, ^13^, ^14^] bio‐labeling/bio‐imaging[^15^, ^16^] and in various other applications.[^17^, ^18^, ^19^, ^20^, ^21^] It is also frequently used in conjugates with various drugs,[^22^, ^23^, ^24^, ^25^] nanoparticles,[^26^, ^27^] or proteins.[^18^, ^28^] The application of BODIPY dyes in medical research and chemical biology studies was nicely reviewed by Marfin et al. in 2017.^[29]^

Very recently, a model fluorescent system able to reflect the enhanced concentration of glutathione causing the drug release has been described by our group.^[30]^ This new drug‐delivery system is based on 3‐hydroxyquinolin‐4(1*H*)‐ones (3‐HQ) as a model drug conjugation with fluorescent amino‐BODIPY dye enabling the tracking of the whole system and detection of the drug release. Importantly, the drug and the dye are connected through a self‐immolative disulfide linker allowing its selective cleavage inside a cancer cell.[^31^, ^32^] This phenomenon is possible due to glutathione (GSH) as a linker cleavage agent. Its concentration in cancer cells, reaching up to 10 mm,[^33^, ^34^] is by 2–3 orders of magnitude higher than in plasma and blood.[^35^, ^36^]

According to numerous previously published studies, the BODIPY dyes are used as the fluorescent species with high intensity and low toxicity.^[29]^ Although many of the recently developed BODIPY‐drug conjugates[^37^, ^38^, ^39^, ^40^, ^41^, ^42^] indicate the BODIPY as a promising candidate for biological applications, to the best of our knowledge, none of the studies describe its potential cytotoxicity or even direct application as an cytotoxic agent.

Here we report the amino‐BODIPY dye as an anticancer agent. Its cytotoxicity is possible to modulate via conjugation with 3‐HQs to achieve selective cytotoxicity against leukemia cell lines.

## 2 Results and Discussion

**Figure 1:**
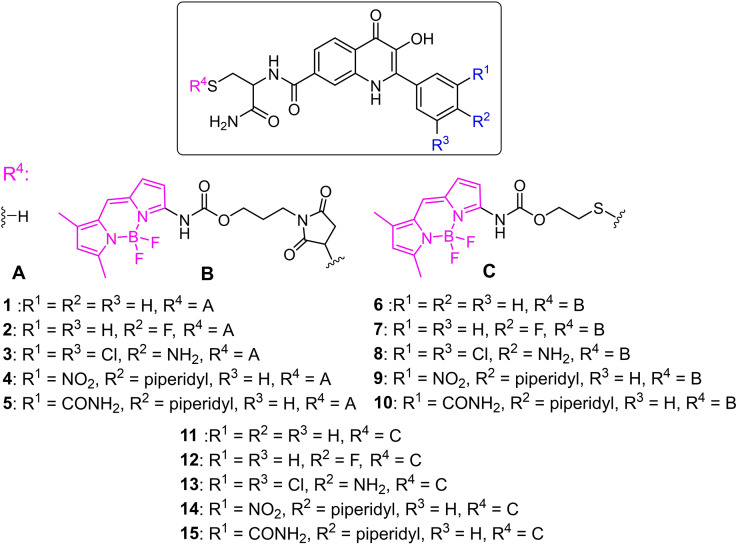
Prepared and studied 3‐HQ derivatives **1–15**

For the amino‐BODIPY conjugation, we selected and synthesized five 3‐HQ derivatives **1–5** (Figure 1) with different substitutions on the 2‐phenyl ring as the counterparts. Compound **1** is derived from 2‐phenyl‐3‐hydroxy‐4(1*H*)‐quinolinone reported to have no activity against cancer cells.^[43]^ The derivative **2** is derived from the 2‐(4‐fluorophenyl)‐3‐hydroxy‐4(1*H*)‐quinolinone with low cytotoxicity against cancer cells.^[44]^ The compound **3** comes from a modification of 2‐(4‐amino‐3,5‐dichlorophenyl)‐3‐hydroxy‐4(1*H*)‐quinolinone reported previously as the active derivative against various cancer cell lines. Product **4** was prepared by modification of the 2‐(3‐nitro‐4‐piperid‐1‐ylphenyl)‐3‐hydroxy‐4(1*H*)‐quinolinone having significant activity but low selectivity against cancer cell lines.^[45]^ Compound **5** is then derived from active 2‐(3‐carboxamide‐4‐piperid‐1‐ylphenyl)‐3‐hydroxy‐4(1*H*)‐quinolinone with significant activity and increased selectivity toward cancer cells.^[46]^ Carboxamide moiety with lipophilic *N*‐substitution in position 7 was previously reported as the substituent co‐responsible for cytotoxic activity when combined with suitable substituents on the 2‐phenyl ring.^[47]^ The substituted carboxamide effect is possibly connected with the ability of compounds to penetrate through the cell membrane. We have implemented cysteine‐amide to position 7 of the quinolinone framework for our studies to enable the conjugation with the amino‐BODIPY dye **16**. Thus, these compounds **1–5** were then incorporated into two sets of conjugates bearing amino‐BODIPY. Conjugates **6–10** include maleimide linker, which is expected to be uncleavable under GSH treatment. Contrary, conjugates **11–15** contain the disulfide linker sensitive to GSH cleavage followed by drug/dye liberation as described previously.^[30]^

### 2.1 Synthesis

**Scheme 1:**
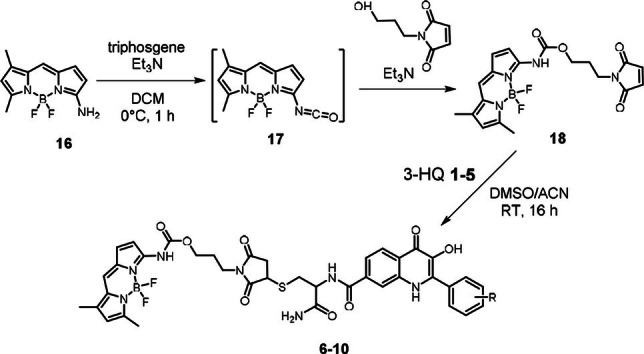
Synthesis of maleimide conjugates **6–10**. For the structure of the compounds **1–5** and the substituents R see Figure 1.

The synthetic approach to compounds **1–5** and **11–15** was inspired by the described procedure^[30]^ (for details, see Experimental part). The conjugates **6–10** were prepared according to the following Scheme 1. Amino‐BODIPY **16** was transformed to isocyanate **17** followed by reaction with 1‐(3‐hydroxypropyl)‐1*H*‐pyrrole‐2,5‐dione to obtain derivative **18**. It was then reacted with 3‐HQs **1–5** to get a targeted set of BODIPY‐3HQs conjugates **6–10**.

**Figure 2:**
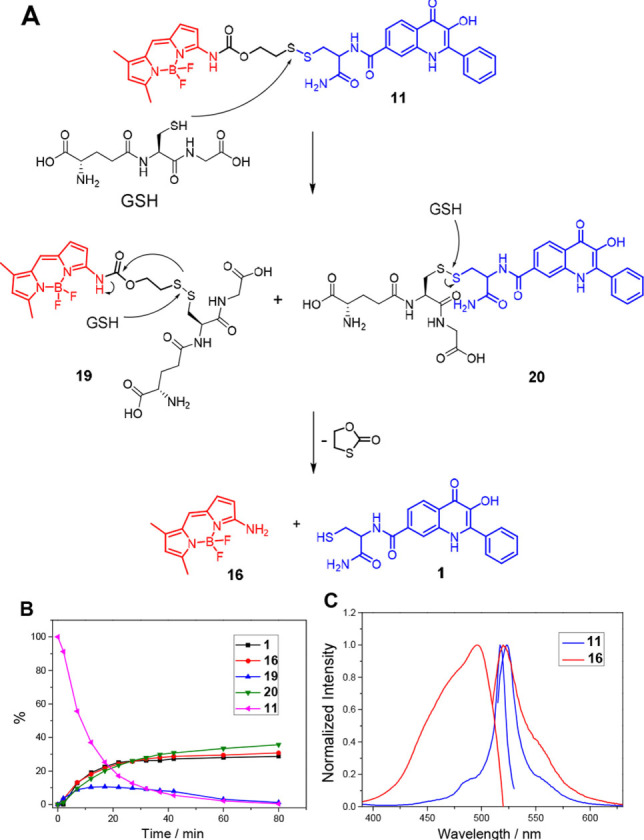
(A) Mechanism of GSH‐mediated cleavage of conjugate **11** (5 mm GSH, 5 μM conjugate **11** in HEPES buffer, 7.4 pH, 37 °C). (B) Time‐dependent stability of compounds **1**, **11**, **16**, **19** and **20** monitored by LC/MS. (C) Excitation/emission spectra of the conjugate **11** and the released Amino‐BODIPY **16** enabling the OFF‐ON fluorescence effect when excitation at 485 nm and emission at 530 nm is applied.

As reported previously, the GSH mediated cleavage of the disulfide linker results in the release of the 3‐HQ derivative together with the Amino‐BODIPY.^[30]^ Different excitation and very similar emission profile of the free Amino‐BODIPY **16** and the one bound in the conjugates enabling the OFF‐ON effect is demonstrated for conjugate **11** in Figure 2, where excitation and emission spectra of compounds **16** and **11** are presented. As the mechanism of GSH‐mediated cleavage and LC/MS analysis in Figure 2A and 2B depict nucleophilic thiol group on glutathione attacks the disulfide bond resulting in the formation of GSH adducts **19** and **20**. Intermediate **19** further reacts with excess of GSH and self‐immolative linker is cyclized while free Amino‐BODIPY **16** is released. According to LC/MS analysis the intermediate **20** is relatively stable and further conversion to free 3‐HQ **1** was not observed.

**Figure 3:**
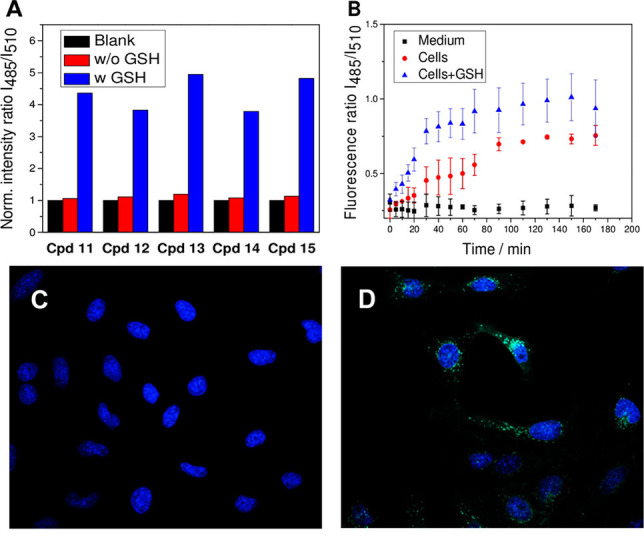
(A) Schematic representation of the ratiometric change of fluorescence intensities at 530 nm after excitation at 485 nm and 510 nm (Fluorescence ratio I_485_/I_510_) for conjugates **11–15** incubated in HEPES buffer and measured at 0 h (black columns), HEPES buffer without GSH for 3 h (red columns) and in HEPES buffer with GSH (5 mm) for 3 h at 37 °C (blue columns). (B) Time monitored cleavage of conjugate **13** in medium without GSH (black), after treatment of the HeLa cells (red) or HeLa cells pretreated by 20 mm GSH (blue) with conjugate **13**. (C) The microscopy images of the internalization of conjugate **13** inside the HeLa cells before treatment and (D) 2 h after treatment with GSH (20 mm).

To evaluate the cleavability of conjugates **6–15**, their fluorescence spectra were measured in HEPES buffer with and without the presence of GSH (5 mm). The cleavable conjugates **11–15** have an emission maximum at around 530 nm after excitation by 510 nm. Their cleavage affords the amino‐BODIPY **16** with the similar emission maximum (530 nm) achieved after excitation at different wavelength (485 nm). Thus, when the ratio of emission intensities at 530 nm obtained after excitation at 485 nm and 510 nm was monitored within the time, the total conjugate cleavage was possible to detect by ratiometric fluorescence sensing (see Figure 3). As demonstrated in Figure 3A, conjugates **11–15** exhibit sufficient stability within the first three hours of the experiment when dissolved in HEPES buffer in the absence of GSH. When GSH as the cleavage agent is added, the Amino‐BODIPY **16** releasing accompanied by the 3‐HQs detachment^[30]^ is indicated by a substantial increase of the 530 nm emission intensity ratio obtained after 485 nm and 510 nm excitation (I_485_/I_510_) (Figure 3A).

### 2.2 Study of Conjugate Cleavage Inside Cells

Precise time monitoring of the drug release was performed in HeLa cells, where the conjugate was disrupted to a maximal level within the first several tens of minutes as demonstrated in Figure 3B and Figures S1‐S3 in the Supporting Information. When the HeLa cells were pretreated by glutathione to increase the internal concentration of thiol, the cleavage was faster. The value of I_485_/I_510_ responding to the amino‐BODIPY, and drug release responded to a higher concentration of these liberated compounds.

Additionally, HeLa cells were treated with these conjugates, and microscopy images of their cellular internalization before and after treatment with GSH (20 mm) were recorded. It is apparent that after GSH treatment, the green fluorescence of released Amino‐BODIPY **16** has appeared. Thus OFF‐ON effect is observed as exemplified in Figures 3C and 3D.

**Figure 4:**
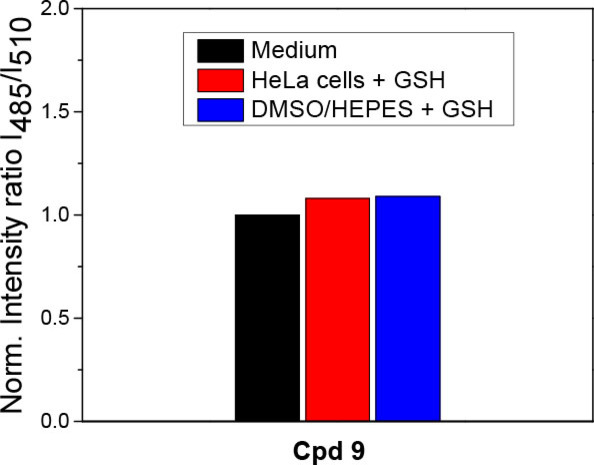
Ratio of fluorescence intensities at 530 nm after excitation at 485 nm and 510 nm (I_485_/I_510_) after 180 min. incubation of non‐cleavable conjugate **9** (5 μM) in free medium (black column), HeLa cells with additional GSH (20 mm) (red column) and in the presence of GSH (5 mm) in DMSO/HEPES buffer 2 : 1 (0.1 M, pH 7.4) (blue column). All experiments were carried out upon incubation at 37 °C.

Similarly, fluorescence ratio I_485_/I_510_ of non‐cleavable conjugates **6‐10** was monitored in DMSO/HEPES buffer (2 : 1) (Figure 4, Figure S4). In these cases, no significant changes were observed, confirming the conjugates′ inertness towards the GSH. The conjugates are also stable in HeLa cells, as demonstrated on representative derivative **9** (Figure 4).

### 2.3 Cytotoxic Activity

Finally, the amino‐BODIPY **16** and all prepared conjugates **1–15** were tested for cytotoxic activity against selected cancer cell lines (Table 1). The tests were performed on cancer cell lines derived from solid tumors as well as hematological malignancies: CCRF‐CEM (acute lymphoblastic leukemia), K562 (chronic myeloid leukemia), A549 (lung adenocarcinoma), colorectal carcinoma cell lines HCT116 with and without functional p53 protein HCT116p53, respectively. The panel also included chemoresistant subclone CCRF‐CEM‐DNR (resistant to daunorubicine) overexpressing P‐glycoprotein and/or lung resistance‐related protein (LRP), which are pumps or detoxifying systems responsible for the most common forms of clinical resistance. To evaluate non‐tumor cells′ toxicity, we used human skin fibroblast cell line BJ and lung fibroblast cell line MRC‐5. From Table 1, we can see that non‐conjugated 3‐HQs (**1**‐**5**) do not exhibit any cytotoxic activity, while amino‐BODIPY **16** is active against lymphoblastic as well as myeloid leukemia cell lines and also against colorectal carcinoma. This dye is also slightly toxic to normal fibroblast BJ and MRC with IC_50_>40 μM and low‐density seeding variants BJ‐LD and MRC‐LD with higher proliferation and no contact inhibition. Connection of amino‐BODIPY **16** with 3‐HQs via non‐cleavable maleimide linker (conjugates **6–10**) as well as via cleavable linker (compounds **11–15**) causes higher selectivity toward CCRF‐CEM lines. The exception is derivative **9** having the selectivity to K562 line and derivative **15**, which is entirely inactive, probably due to low solubility. Contrary to the free Amino‐BODIPY **16** none of the conjugates exhibit toxicity against the BJ, MRC, BJ‐LD, or MRC‐LD lines, suggesting the selectivity of conjugates to cancer cells. According to these results, we can conclude that the conjugation of cytotoxic Amino‐BODIPY and inactive 3‐HQs alters its cytotoxicity profile and gives the selectivity to leukemia cell lines. This effect is surprisingly independent of the cleavability of conjugates, what can be explained by the ability of pharmacophore to interact with a target regardless of release from the conjugate. The selectivity of conjugates toward leukemia cells could be caused by interaction with a specific target for the CCRF‐CEM or K562 cells, respectively, or by particular transport to these cell lines. The later reason could explain the lower toxicity of amino‐BODIPY released from the conjugate compared to free amino‐BODIPY **16** directly applied to the cells.

**Table 1:** Cytotoxic activities of prepared compounds (IC_50_ [μm]).

Cmp. N^o^	CCRF‐CEM	CEM‐DNR	K562	K562‐TAX	A549	HCT116	HCT116p53	U2OS	BJ	BJ‐LD	MRC	MRC‐LD
**1‐5**	>50	>50	>50	>50	>50	>50	>50	>50	>50	ND	>50	ND
**6**	14.60	>50	>50	>50	>50	>50	>50	>50	>50	>50	>50	>50
**7**	6.37	>50	>50	>50	>50	>50	>50	>50	>50	>50	>50	>50
**8**	12.70	>50	>50	>50	>50	>50	>50	>50	>50	>50	>50	>50
**9**	>50	>50	4.05	>50	>50	>50	>50	>50	>50	ND	>50	ND
**10**	5.10	>50	>50	49.55	>50	>50	>50	>50	>50	>50	>50	>50
**11**	19.73	34.42	49.82	39.77	49.89	>50	>50	>50	>50	>50	>50	>50
**12**	8.14	26.51	44.65	33.59	46.43	>50	49.79	42.30	>50	>50	>50	>50
**13**	2.59	40.09	41.43	41.83	>50	>50	>50	>50	>50	>50	>50	>50
**14**	1.52	37.91	29.91	40.61	46.27	39.71	42.60	44.15	>50	>50	>50	>50
**15**	>50	46.25	>50	46.16	>50	>50	>50	>50	>50	>50	>50	>50
**16**	4.25	5.08	3.29	6.41	47.2	11.56	12.74	19.80	49.66	28.10	40.11	26.94

[a] Average values of IC_50_ from at least three independent experiments with SD ranging from 1 to 20 % of the average values.

## 3 Conclusion

A series of target conjugates were synthesized by combining 2‐phenyl‐3‐hydroxy‐4(1*H*)‐quinolinone (3‐HQ) derivatives with Amino‐BODIPY dye. While some of them (**6‐10**) were uncleavable in the presence of glutathione in increased concentration, disruption of cleavable conjugates (**11‐15**) within the time was possible to monitor using ratiometric fluorescence. The released Amino‐BODIPY is possible to detect also by the OFF‐ON effect. While the prepared 3‐HQs appeared to be quite inactive to selected cancer cell lines, the Amino‐BODIPY was proved to possess cytotoxic activity against almost all of them as well as against proliferating non‐tumor cells. When these cell lines were treated with Amino‐BODIPY conjugated with 3HQs, the selectivity against lymphoblastic or myeloid leukemia has appeared. The cytotoxicity of the conjugates against normal cells has disappeared regardless of the linker cleavability. The specific toxicity of the system to leukemia cells and a possibility of a synergic effect of the Amino‐BODIPY and maybe any other anticancer agent accompanied by a possibility of the cleavage monitoring could make this system attractive for future studies of new theranostics.

## Experimental Section

### Materials and Methods

All chemicals and solvents for the synthesis were obtained from Sigma‐Aldrich. NMR spectra were measured in DMSO‐*d_6_* and CDCl_3_ using a Jeol ECX‐500 (500 MHz) spectrometer. Chemical shifts (δ) are reported in parts per million (ppm) and coupling constants (*J*) are reported in Hertz (Hz). HRMS analysis was performed using an Exactive Plus Orbitrap high‐resolution (Thermo Fischer Scientific, MA, USA). The machine was operated at the positive full scan mode (120 000 FWMH). The chromatographic separation was performed using column Phenomenex Gemini (C18, 50×2 mm, 3 μm particles) in isocratic mode with mobile phase using 95 % MeOH and 5 % H_2_O with 0.1 % of formic acid.

### Cleavage of Conjugates 6‐10 and 11‐15 by Glutathione and its Fluorescence Monitoring

First, 5 μl of the solution of conjugates **6–10** and **11–15** (1 mm) in DMSO was mixed with 20 μl, 60 μl or 100 μl of the GSH solution (50 mm) in HEPES buffer (0.1 M; pH 7.4) and diluted with HEPES buffer (0.1 M; pH 7.4) or DMSO/HEPES buffer (2 : 1) to 1 ml. The mixture was heated to 37 °C, and the fluorescence was measured in time. after

### Intracellular Cleavage of Conjugates 6‐10 and 11‐15 by Glutathione and its Fluorescence Monitoring

HeLa cells were added to blank 96‐well plates by MultiDrop Combi (Thermo Fisher Scientific, USA) at a cell density of 1.25×104 per well and incubated overnight. The pretreatment with GSH was performed by the incubation of cells with GSH (20 mm in medium) for 2 h. The cells were washed with PBS, immediately treated with the tested compounds for 2 min at 37 °C and washed with PBS again. Finally, 50 μL of PBS was added to each well. The fluorescence intensity was measured by an EnVision plate reader (Perkin Elmer, USA), with two reads for each time point (first, with ex 510 nm/em 535 nm, and second, with ex 485 nm/em 535 nm).

### Quantum Yield Determination

Quantum yields (Φ) were calculated by the standard procedure using fluorescein in 0.1M NaOH as a reference (Φ=0.91) and according to equation 1.

(1) $$\Phi =\Phi {}_{R}\times I/I{}_{R}\times A{}_{R}/A\times \eta \;2/\left(\eta {}_{R}\;2\right);$$

where Φ_R_ is the quantum yield of the reference fluorophore, I is the area under the emission peak, A is absorbance at the excitation wavelength, and η is the refractive index of the solvent.

### Synthesis of BODIPY Conjugates

The compounds **1–5** were prepared by solid‐phase chemistry approach according to the published procedure.^[30]^

### Characterization of compound 1 was in accordance with the published data^[30]^

**1**: ^1^H NMR (500 MHz, DMSO‐*d_6_*) δ 11.82 (bs, 1H), 8.60 (d, *J*=7.9 Hz, 1H), 8.26 (s, 1H), 8.22 (d, *J*=8.50 Hz, 1H), 7.82 (d, *J=*7.2 Hz, 2H), 7.76 (dd, *J=*1.2, 8.5 Hz, 1H), 7.50–7.60 (m, 4H), 7.22 (s, 1H), 4.50‐4.58 (m, 1H), 2.94–3.03 (m, 1H), 2.82–2.93 (m, 1H), 2.39 (t, *J=* 8.4 Hz, 1H) ^13^C NMR δ=171.69, 169.48, 166.09, 138.55, 137.38, 135.93, 132.64, 132.09, 129.03, 129.28, 128.31, 124.59, 123.09, 120.26, 118.90, 55.97, 26.00. HRMS (ESI) m/z calcd for C_19_H_17_N_3_O_4_S^+^ [M+H]^+^: 383.0940; found: 383.0944. Yield: 95 %. Obtained as a light‐yellow solid.

**2**: ^1^H NMR (500 MHz, DMSO‐*d_6_*) δ 11.80 (s, 1H), 8.60 (d, *J*=7.9 Hz, 1H), 8.25 (s, 1H), 8.22 (d, *J*=8.5 Hz, 1H), 7.89 (dd, *J*=8.4, 5.6 Hz, 2H), 7.76 (d, *J*=8.6 Hz, 1H), 7.55 (s, 1H), 7.42 (t, *J*=8.8 Hz, 2H), 7.22 (s, 1H), 4.58‐4.50 (m, 1H), 2.98 (dd, *J*=8.7, 4.7 Hz, 1H), 2.89 (dd, *J*=15.1, 6.9 Hz, 1H), 2.39 (t, *J*=8.4 Hz, 1H). ^13^C NMR (50 MHz, DMSO‐d_6_) δ 171.66, 166.05, 163.49, 161.52, 138.52, 137.39, 135.94, 131.67, 131.60, 124.61, 123.13, 120.21, 115.37, 115.20, 55.94, 25.99. HRMS (ESI) m/z calcd. for C_19_H_17_FN_3_O_4_S^+^ [M+H]^+^: 402.0918, found: 402.0920. Yield: 92 %. Obtained as a light‐yellow solid.

**3**: ^1^H NMR (500 MHz, DMSO‐*d_6_*) δ 11.62 (s, 1H), 8.58 (t, *J*=7.9 Hz, 1H), 8.27 (d, *J*=1.1 Hz, 1H), 8.20 (t, *J*=8.2 Hz, 1H), 7.84 (d, *J*=12.7 Hz, 2H), 7.75 (dd, *J*=8.6, 1.4 Hz, 1H), 7.54 (s, 1H), 7.22 (s, 1H), 4.59–4.52 (m, 1H), 3.02–2.95 (m, 1H), 2.91‐2.86 (m, 1H), 2.54 (s, 2H), 2.39 (t, *J*=8.4 Hz, 1H). ^13^C NMR (50 MHz, DMSO‐d_6_) δ 171.66, 166.05, 142.14, 138.32, 137.34, 135.81, 128.66, 124.49, 122.92, 120.17, 119.92, 117.44, 55.94, 25.99. HRMS (ESI) m/z calcd. for C_19_H_17_Cl_2_N_4_O_4_S^+^ [M+H]^+^: 467.0342, found: 467.0344. Yield: 89 %. Obtained as a yellow‐greenish solid.

Characterization of the compound **4** was in accordance with the published data.^[30]^

**4**: ^1^H NMR (500 MHz, DMSO‐*d_6_*) δ 11.74 (bs, 1H), 8.61 (d, J= 7.9 Hz, 1H), 8.30 (s,1H), 8.25 (s, 1H), 8.21 (d, J= 8.5 Hz, 1H), 8.03 (d, J= 8.5 Hz, 1H), 7.75 (d, J= 8.5 Hz, 1H), 7.54 (s, 1H), 7.43 (d, J= 8.8 Hz, 1H), 7.22 (s, 1H), 4.54 (dd, J= 8.3, 12.7 Hz, 1H), 3.10 (s, 4H), 2.95–3.03 (m, 1H), 2.83–2.92 (m, 1H), 2.39 (t, J= 8.4 Hz, 1H), 1.60‐1.64 (s, 6H). ^13^C NMR δ= 172.20, 170.13, 166.58, 145.75, 140.80, 139.22, 137.95, 136.50, 134.83, 130.69, 127.19, 125.15, 123.67, 123.56, 120.88, 120.75, 119.28, 56.51, 52.29, 26.52, 25.91, 23.93. HRMS (ESI) m/z calcd for C_24_H_25_N_5_O_6_S^+^ [M+H]^+^: 512.1598; found: 512.1608. Yield: 94 %. Obtained as an orange solid.

**5**: ^1^H NMR (500 MHz, DMSO‐*d_6_*) δ 11.82 (s, 1H), 8.60 (d, *J*=8.0 Hz, 1H), 8.56 (s, 1H), 8.26 (dd, *J*=11.8, 1.7 Hz, 2H), 8.22 (d, *J*=8.6 Hz, 1H), 7.95 (dd, *J*=8.4, 2.2 Hz, 1H), 7.79 (s, 1H), 7.75 (dd, *J*=8.6, 1.5 Hz, 1H), 7.54 (s, 1H), 7.46 (d, *J*=8.3 Hz, 1H), 7.21 (s, 1H), 4.54 (td, *J*=8.5, 4.7 Hz, 1H), 3.12–3.05 (m, 4H), 3.02–2.96 (m, 1H), 2.92–2.85 (m, 1H), 2.39 (t, *J*=8.4 Hz, 1H), 1.88 (s, 1H), 1.77–1.71 (m, 4H), 1.61–1.56 (m, 2H). ^13^C NMR (50 MHz, DMSO‐d_6_) δ 171.80, 171.66, 169.39, 168.21, 166.09, 158.22, 157.94, 138.56, 137.40, 135.91, 132.73, 131.66, 130.95, 127.35, 124.58, 123.09, 120.23, 119.69, 118.79, 55.97, 54.73, 53.83, 26.10, 25.99, 25.59, 22.97, 22.56. HRMS (ESI) m/z calcd. for C_25_H_28_N_5_O_5_S^+^ [M+H]^+^: 510.1806, found: 510.1803. Yield: 62 %. Obtained as a light‐yellow solid.

**18**: Solution of amino‐BODIPY **16** (258 mg, 1.098 mmol) and triethylamine (160 μL, 1.153 mmol) in DCM (3 mL) was added to the solution of triphosgene (108 mg, 0.362 mmol) while cooled to 0 °C. After stirring for 1 h at 0 °C the solution of *N*‐(3‐hydroxypropyl)maleimide (170 mg, 1.098 mmol) and triethylamine (160 μL, 1.153 mmol) in DCM (3 mL) was added slowly. Reaction mixture was then warmed to RT and stirred for 16 h. Ethylacetate (100 mL) was added to the reaction mixture and it was washed with water and brine 3 times. Organic layer was dried over anhydrous sodium sulfate and concentrated under reduced pressure. Crude product was purified by column chromatography (hexane/ethylacetate 2 : 1) to obtain 240 mg (53 %) of pure compound. ^1^H NMR (500 MHz, CDCl_3_) δ 7.97 (s, 1H), 7.00 (s, 1H), 6.99 (d, *J*=4.5 Hz, 1H), 6.88 (d, *J*=4.4 Hz, 1H), 6.73 (s, 2H), 6.04 (s, 1H), 4.23 (t, *J*=6.2 Hz, 2H), 3.68 (t, *J*=6.7 Hz, 2H), 2.52 (s, 3H), 2.22 (s, 3H), 2.02 (p, *J*=6.5 Hz, 2H). ^13^C NMR (50 MHz, CDCl_3_) δ 170.82, 155.43, 151.61, 149.94, 141.05, 134.43, 133.58, 131.03, 129.86, 122.03, 118.89, 109.44, 64.23, 35.14, 27.72, 14.67, 11.38. MS (ESI) m/z calcd. for C_19_H_20_BF_2_N_4_O_4_^+^ [M+H]^+^: 417.15; found: 417.50. Obtained as a dark red solid.

**Preparation of Compounds 6–10**: Starting compound **18** (30 mg, 0.072 mmol) in DMSO/MeCN (1 : 1, v/v, 1 mL) was mixed with triethylamine (50 μL, 0.360 mmol) and corresponding 3‐HQ **2**, **3** or **5** (0.072 mmol). The resulting mixture was stirred 16 h at RT. Product was then extracted with EtOAc (50 mL) and washed with water and brine 3 times. Organic layer was dried over anhydrous sodium sulphate and concentrated under reduced pressure to give 45 mg (78 %) of crude product. Purification by column chromatography (DCM/MeOH gradient) or by HPLC (CH_3_COONH_4_/MeCN) was performed for all final compounds.

**6**: ^1^H NMR (500 MHz, DMSO‐d_6_) δ 11.80 (s, 1H), 8.75 (d, *J*=8.1 Hz, 1H), 8.27 (s, 1H), 8.23 (d, *J*=8.5 Hz, 1H), 7.83 (d, *J*=7.3 Hz, 2H), 7.75 (d, *J*=8.6 Hz, 1H), 7.61–7.49 (m, 5H), 7.30–7.21 (m, 2H), 6.80 (t, *J*=4.5 Hz, 1H), 6.18 (s, 1H), 4.73–4.65 (m, 1H), 4.16 (t, *J*=5.7 Hz, 2H), 4.13–4.07 (m, 1H), 3.54 (t, *J*=6.7 Hz, 2H), 3.37 (dd, *J*=13.3, 4.5 Hz, 1H), 3.27–3.19 (m, 2H), 3.06 (dd, *J*=13.3, 9.9 Hz, 1H), 2.62–2.54 (m, 1H), 2.42 (s, 3H), 2.22 (s, 3H), 1.91–1.87 (m, 2H). ^13^C NMR (50 MHz, DMSO‐d_6_) δ 176.83, 176.70, 175.14, 172.01, 171.75, 171.70, 169.66, 166.09, 165.94, 151.72, 138.60, 137.43, 135.81, 135.77, 132.86, 132.39, 132.12, 131.73, 129.58, 129.37, 129.26, 128.27, 124.62, 123.12, 120.10, 118.94, 118.67, 109.39, 63.93, 63.87, 52.97, 52.80, 48.61, 36.05, 35.87, 35.28, 32.97, 26.37, 26.35, 21.03, 14.17, 10.91. HRMS (ESI) m/z calcd. for C_38_H_37_BF_2_N_7_O_8_S^+^ [M+H]^+^: 800.2480, found: 800.2489. Yield: 34 %. Φ_F_=0.24. Obtained as a dark red solid.

**7**: ^1^H NMR (500 MHz, DMSO‐d_6_) δ 11.80 (s, 1H), 8.75 (d, *J*=8.0 Hz, 1H), 8.25 (s, 1H), 8.22 (d, *J*=8.5 Hz, 1H), 7.88 (dd, *J*=8.3, 5.7 Hz, 2H), 7.75 (d, *J*=8.6 Hz, 1H), 7.57 (dd, *J*=10.4, 6.1 Hz, 2H), 7.41 (t, *J*=8.5 Hz, 2H), 7.25 (d, *J*=6.0 Hz, 2H), 6.80 (t, *J*=4.7 Hz, 1H), 6.19 (s, 1H), 4.73–4.65 (m, 1H), 4.16 (t, *J*=5.7 Hz, 2H), 4.10 (ddd, *J*=9.0, 7.1, 3.9 Hz, 1H), 3.54 (t, *J*=6.7 Hz, 2H), 3.37 (dd, *J*=13.3, 4.4 Hz, 1H), 3.23 (dd, *J*=18.3, 8.8 Hz, 2H), 3.06 (dd, *J*=13.3, 10.0 Hz, 1H), 2.62–2.55 (m, 1H), 2.42 (s, 3H), 2.22 (s, 3H), 1.93–1.87 (m, 3H). ^13^C NMR (50 MHz, DMSO‐d_6_) δ 176.82, 176.70, 175.14, 175.13, 172.00, 171.75, 171.70, 169.66, 166.06, 165.90, 163.49, 161.52, 154.45, 151.61, 149.43, 141.11, 138.56, 137.41, 135.83, 132.90, 131.85, 131.82, 131.80, 131.78, 131.73, 131.68, 131.61, 131.55, 131.39, 129.52, 128.47, 124.63, 123.36, 123.16, 120.13, 118.90, 118.74, 115.35, 115.18, 109.30, 63.95, 63.89, 54.89, 52.95, 52.80, 26.38, 26.35, 21.03, 14.17, 14.16, 10.91. HRMS (ESI) m/z calcd. for C_38_H_36_BF_3_N_7_O_8_S^+^ [M+H]^+^: 818.2386, found: 818.2397. Yield: 35 %. Φ_F_=0.25. Obtained as a dark red solid.

**8**: ^1^H NMR (500 MHz, DMSO‐d_6_) δ 8.74 (d, *J*=8.2 Hz, 1H), 8.26 (s, 1H), 8.18 (dd, *J*=8.5, 1.8 Hz, 1H), 7.82 (s, 2H), 7.73 (dd, *J*=8.6, 1.3 Hz, 1H), 7.60–7.51 (m, 1H), 7.27–7.21 (m, 2H), 6.79 (dd, *J*=6.0, 4.4 Hz, 1H), 6.17 (s, 1H), 6.01 (s, 2H), 4.73–4.64 (m, 1H), 4.18–4.13 (m, 2H), 4.12–4.07 (m, 1H), 3.54 (t, *J*=6.7 Hz, 2H), 3.36 (dd, *J*=13.3, 4.5 Hz, 1H), 3.27–3.19 (m, 2H), 3.06 (dd, *J*=13.3, 9.9 Hz, 1H), 2.62–2.53 (m, 1H), 2.41 (s, 3H), 2.21 (s, 3H), 1.95–1.86 (m, 4H). ^13^C NMR (50 MHz, DMSO‐d_6_) δ 176.83, 176.71, 175.15, 175.14, 172.01, 171.74, 171.69, 166.06, 165.91, 142.11, 138.37, 137.38, 135.69, 132.77, 131.73, 129.68, 128.65, 124.51, 122.95, 120.06, 118.87, 118.50, 117.45, 109.56, 63.88, 52.94, 52.79, 48.60, 26.35, 21.05, 14.16, 10.90. HRMS (ESI) m/z calcd. for C_38_H_36_BCl_2_F_2_N_8_O_8_S^+^ [M+H]^+^: 883.1810, found: 883.1821. Yield: 29 %. Φ_F_=0.19. Obtained as a dark red solid.

**9**: ^1^H NMR (500 MHz, DMSO‐d_6_) δ 8.76 (d, *J*=8.1 Hz, 1H), 8.30 (d, *J*=1.9 Hz, 1H), 8.25 (s, 1H), 8.20 (dd, *J*=8.5, 1.8 Hz, 1H), 8.02 (d, *J*=8.5 Hz, 1H), 7.74 (d, *J*=8.6 Hz, 1H), 7.56 (dd, *J*=17.8, 5.6 Hz, 2H), 7.41 (dd, *J*=8.9, 1.7 Hz, 1H), 7.24 (dd, *J*=9.3, 4.2 Hz, 2H), 6.79 (dd, *J*=6.3, 4.4 Hz, 1H), 6.18 (s, 1H), 4.73–4.64 (m, 1H), 4.15 (t, *J*=6.0 Hz, 2H), 4.12–4.07 (m, 1H), 3.54 (t, *J*=6.7 Hz, 2H), 3.36 (dd, *J*=13.3, 4.4 Hz, 1H), 3.24 (d, *J*=9.0 Hz, 1H), 3.21 (d, *J*=9.1 Hz, 1H), 3.11–3.07 (m, 4H), 2.61–2.53 (m, 1H), 2.41 (s, 3H), 2.21 (s, 3H), 1.93–1.86 (m, 3H), 1.67–1.61 (m, 4H), 1.61–1.56 (m, 2H). ^13^C NMR (50 MHz, DMSO‐d_6_) δ 176.82, 176.70, 175.13, 175.12, 171.99, 171.73, 171.69, 171.05, 166.04, 165.89, 146.20, 140.26, 138.69, 134.52, 134.27, 132.84, 131.73, 129.58, 126.64, 124.59, 123.13, 120.30, 120.15, 118.63, 109.42, 63.91, 63.86, 56.03, 54.89, 52.95, 52.80, 51.75, 48.59, 26.37, 26.34, 25.37, 23.38, 21.03, 18.53, 14.16, 10.91, 10.90. HRMS (ESI) m/z calcd. for C_43_H_45_BF_2_N_9_O_10_S^+^ [M+H]^+^: 926.2920, found: 926.2941. Yield: 44 %. Φ_F_=0.05. Obtained as a dark red solid.

**10**: ^1^H NMR (500 MHz, DMSO‐d_6_) δ 11.77 (s, 1H), 8.76 (d, *J*=8.0 Hz, 1H), 8.49 (s, 1H), 8.27 (s, 1H), 8.21 (dd, *J*=8.7, 1.4 Hz, 2H), 7.90 (d, *J*=8.2 Hz, 1H), 7.73 (d, *J*=8.6 Hz, 1H), 7.64 (s, 1H), 7.61–7.52 (m, 2H), 7.34–7.29 (m, 1H), 7.28–7.20 (m, 2H), 6.80 (t, *J*=4.5 Hz, 1H), 6.18 (s, 1H), 4.73–4.64 (m, 1H), 4.16 (t, *J*=5.8 Hz, 2H), 4.10 (ddd, *J*=9.1, 6.5, 3.9 Hz, 1H), 4.10 (ddd, *J*=9.1, 6.5, 3.9 Hz, 1H), 3.54 (t, *J*=6.7 Hz, 2H), 3.37 (dd, *J*=13.3, 4.4 Hz, 1H), 3.24 (d, *J*=8.8 Hz, 1H), 3.21 (d, *J*=8.8 Hz, 1H), 3.06 (dd, *J*=13.3, 10.0 Hz, 1H), 3.01–2.94 (m, 4H), 2.61–2.54 (m, 1H), 2.42 (s, 3H), 2.22 (s, 3H), 1.93–1.87 (m, 3H), 1.70 (s, 4H), 1.58–1.51 (m, 2H). ^13^C NMR (50 MHz, DMSO‐d_6_) δ 176.83, 176.71, 175.15, 175.13, 172.01, 171.77, 171.72, 168.02, 166.14, 165.99, 154.43, 152.66, 151.63, 149.46, 141.08, 138.56, 137.42, 135.77, 132.90, 132.35, 131.73, 131.13, 129.53, 128.12, 125.84, 124.58, 123.34, 123.11, 120.10, 120.08, 119.20, 118.87, 118.73, 109.31, 63.96, 63.90, 56.05, 53.58, 52.97, 52.81, 48.60, 26.38, 26.35, 25.77, 23.35, 21.04, 18.54, 14.18, 10.91. HRMS (ESI) m/z calcd. for C_44_H_47_BF_2_N_9_O_9_S^+^ [M+H]^+^: 926.3273, found: 926.3285. Yield: 23 %. Φ_F_=0.22. Obtained as a dark red solid.

**Preparation of Compounds 11–15**: The compounds **11–15** were prepared according to the published procedure.^[30]^

**11**: ^1^H NMR (500 MHz, DMSO‐d_6_) δ 11.78 (s, 1H), 8.82 (d, *J*=8.2 Hz, 1H), 8.33 (bs, 1H), 8.25 (d, *J*=1.5 Hz, 1H), 8.21 (d, *J*=8.6 Hz, 1H), 7.80 (m, 2H), 7.74 (dd, *J*=8.6, 1.5 Hz, 1H), 7.62 (bd, *J*=1.5 Hz, 1H), 7.58 (bs, 1H), 7.55 (m, 2H), 7.51 (m, 1H), 7.26 (bd, *J*=1.5 Hz, 1H), 7.21 (d, *J*=4.3 Hz, 1H), 6.71 (d, *J*=4.3 Hz, 1H), 6.21 (s, 1H), 4.73 (ddd, *J*=13.5, 10.2, 4.3 Hz, 1H), 4.41 (m, 2H), 3.29 (dd, *J*=13.5, 4.3 Hz, 1H), 3.13 (dd, *J*=13.5, 10.5, 1H), 3.08 (m, 2H), 2.42 (s, 3H), 2.22 (s, 3H). Hz. ^13^C NMR δ 171.92, 169.84, 166.27, 155.07, 151.45, 149.02, 141.75, 138.77, 137.59, 135.99, 133.22, 132.28, 131.77, 131.51, 129.58, 129.53, 129.45, 128.44, 124.80, 123.89, 123.32, 120.25, 119.10, 119.08, 109.26, 64.02, 52.76, 40.52, 36.04, 14.39, 11.14. HRMS (ESI) m/z calcd. for C_33_H_31_BF_2_N_6_O_6_S_2_^+^ [M+H]^+^: 721.1880; found: 721.1886. Yield: 56 %. Φ_F_=0.16. Obtained as a dark red solid.

**12**: ^1^H NMR (500 MHz, DMSO‐d_6_) δ 11.77 (s, 1H), 8.82 (d, *J*=8.2 Hz, 1H), 8.32 (bs, 1H), 8.24 (d, *J*=8.6 Hz, 1H), 8.20 (d, *J*=8.6 Hz, 1H), 7.86 (m, 2H), 7.74 (dd, *J*=8.6, 1.6 Hz, 1H), 7.62 (bd, *J*=1.5 Hz, 1H), 7.58 (s, 1H), 7.39 (m, 2H), 7.26 (bd, *J*=1.5 Hz, 1H), 7.21 (d, *J*=4.4 Hz, 1H), 6.70 (d, *J*=4.4 Hz, 1H), 6.21 (s, 1H), 4.73 (ddd, *J*=10.2, 8.2, 4.3 Hz, 1H), 4.40 (m, 2H), 3.29 (dd, *J*=13.5, 4.3 Hz, 1H), 3.12 (dd, *J*=13.5, 10.2 Hz, 1H), 3.08 (m, 2H), 2.41 (s, 3H), 2.22 (s, 3H). ^13^C NMR (50 MHz, DMSO‐d_6_) δ 171.94, 169.88, 166.24, 162.67 (*J*_*C‐F*_=247 Hz), 155.07, 151.45, 149.02, 141.75, 138.75, 137.58, 136.01, 133.23, 131.82 (*J*_*C‐F*_=8.6 Hz), 131.80, 131.57, 129.58, 128.63, 123.88, 123.36, 124.83, 120.28, 119.11, 119.08, 115.44 (*J*_*C‐F*_=21.4 Hz), 109.25, 64.03, 52.76, 40.51, 36.01, 14.39, 11.13. MS (ESI) m/z calcd. for C_33_H_31_BF_3_N_6_O_6_S_2_^+^ [M+H]^+^: 739.179; found: 739.253. Yield: 29 %. Φ_F_=0.22. Obtained as a dark red solid.

**13**: ^1^H NMR DMSO‐d_6_ δ=11.55 (s, 1H), 8.83 (d, *J*=8.2 Hz, 1H), 8.29 (bs, 1H), 8.26 (d, *J*=1.7 Hz 1H), 8.17 (d, *J*=8.6 Hz, 1H), 7.80 (s, 2H), 7.74 (dd, *J*=8.6, 1.7 Hz, 1H), 7.63 (bd, *J*=1.5 Hz, 1H) 7.55 (bs, 1H), 7.26 (bd, *J*=1.5 Hz, 1H), 7.18 (d, *J*=4.3 Hz, 1H), 6.67 (d, *J*=4.3 Hz, 1H), 6.19 (s, 1H), 6.02 (bs, 2H), 4.74 (ddd, *J*=10.2, 8.2, 4.2 Hz, 1H), 4.40 (m, 2H), 3.29 (dd, *J*=13.6, 4.2 Hz, 1H), 3.13 (dd, *J*=13.6, 10.2 Hz, 1H), 3.07 (m, 2H), 2.41 (s, 3H), 2.21 (s, 3H). ^13^C NMR (50 MHz, DMSO‐d_6_) δ 171.96. 169.60, 166.23, 155.06, 151.40, 148.96, 142.29, 141.72, 138.59, 137.54, 135.84, 133.22, 131.74, 130.23, 129.55, 128.80, 124.71, 123.82, 123.14, 120.18, 120.09, 119.10, 119.08, 117.61, 109.19, 64.05, 52.74, 40.52, 35.95, 14.11, 11.14. MS (ESI) m/z calcd. for C_33_H_31_BCl_2_F_2_N_7_O_6_S_2_^+^ [M+H]^+^: 804.121; found: 804.419. Yield: 50 %. Φ_F_=0.15. Obtained as a dark red solid.

Characterization of the compound **14** was in accordance with the published data.^[30]^

**14**: ^1^H NMR (500 MHz, DMSO‐d_6_) δ 11.23 (s, 1H), 8.09–7.99 (m, 3H), 7.96 (d, *J*=7.1 Hz, 1H), 7.79 (d, *J*=8.2 Hz, 1H), 7.65 (s, 1H), 7.39 (d, *J*=8.3 Hz, 1H), 7.00 (s, 1H), 6.85 (d, *J*=8.5 Hz, 1H), 6.74–6.70 (m, 1H), 6.67–6.63 (m, 1H), 6.42–6.37 (m, 1H), 6.21 (s, 1H), 5.71 (s, 1H), 4.68–4.58 (m, 1H), 4.18–4.06 (m, 2H), 3.12–2.99 (m, 4.4 Hz, 2H), 2.96–2.87 (m, 2H), 2.79 (s, 4H), 2.72–2.67 (m, 2H), 2.12 (s, 3H), 1.87 (s, 3H), 1.40 (s, 4H), 1.32 (s, 2H). ^13^C NMR δ= 166.77, 166.59, 155.12, 151.11, 149.07, 146.73, 141.81, 141.18, 140.50, 137.70, 135.94, 135.69, 134.10, 133.27, 130.80, 129.38, 127.21, 126.65, 125.00, 122.72, 122.35, 122.11, 120.25, 119.83, 118.83, 118.74, 108.87, 63.99, 52.10, 40.87, 40.77, 36.46, 25.56, 23.69, 14.34, 11.09. HRMS (ESI) m/z calcd for C_38_H_39_BF_2_N_8_O_8_S_2_ [M−H]^−^ : 847.2310; found: 847.2307. Yield: 34 %. Obtained as a dark red solid.

**15**: ^1^H NMR DMSO‐d_6_ δ=11.75 (s, 1H), 8.83 (d, *J*=8.2 Hz, 1H), 8.48 (bd, *J*=3.0 Hz, 1H), 8.26 (d, *J*=1.6 Hz, 3H), 8.19 (d, *J*=8.5 Hz, 1H), 8.18 (d, *J*=2.4 Hz, 1H), 7.87 (s, 1H), 7.73 (dd, *J*=8.5, 1.6 Hz, 1H), 7.66 (bd, *J*=3.0 Hz, 1H), 7.62 (bs, 1H), 7.59 (bs, 1H), 7.31 (d, *J*=8.5 Hz, 1H), 7.26 (bs, 1H), 7.21 (d, *J*=4.3 Hz, 1H), 6.71 (d, *J*=4.3 Hz, 1H), 6.21 (s, 1H), 4.73 (ddd, *J*=13.5, 10.5, 4.3 Hz, 1H), 4.41 (m, 2H), 3.29 (ddd, *J*=13.5, 4.3 Hz, 1H), 3.08 (m, 2H), 2.98 (m, 4H), 2.41 (s, 1H), 2.22 (s, 3H), 1.78 (m, 4H), 1.56 (m, 2H). ^13^C NMR (50 MHz, DMSO‐d_6_) δ 171.93, 169.67, 168.21, 166.32, 155.08, 152.79, 151.46, 149.03, 141.74, 138.73, 137.58, 135.94, 133.22, 132.51, 131.88, 131.78, 131.31, 129.5, 128.26, 125.99, 124.76, 123.89, 123.29, 120.25, 119.35, 119.11, 119.03, 109.28, 64.03, 53.79, 52.78, 40.52, 36.05, 25.90, 23.54, 14.40, 11.14. MS (ESI) m/z calcd. for C_39_H_42_BF_2_N_8_O_7_S_2_^+^ [M+H]^+^: 847.267; found: 847.435. Yield: 43 %. Φ_F_=0.18. Obtained as a dark red solid.

## Conflict of interest

The authors declare no conflict of interest.

## Supporting information

As a service to our authors and readers, this journal provides supporting information supplied by the authors. Such materials are peer reviewed and may be re‐organized for online delivery, but are not copy‐edited or typeset. Technical support issues arising from supporting information (other than missing files) should be addressed to the authors.

Supporting Information
