# Supplementary material for: Cytotoxicity of Amino‐BODIPY Modulated via Conjugation with 2‐Phenyl‐3‐Hydroxy‐4(1H)‐Quinolinones
Source: ChemistryOpen. 2021 Aug 23;10(11):1104–10. doi: 10.1002/open.202100025 (PMC8562313; doi:10.1002/open.202100025)

ChemistryOpen

Supporting Information

Cytotoxicity of Amino-BODIPY Modulated via Conjugation with 2-Phenyl-3-Hydroxy-4(1*H*)-Quinolinones

Martin Porubský, Kristýna Vychodilová, David Milićević, Miloš Buděšinský, Jarmila Stanková, Petr Džubák, Marián Hajdúch, and Jan Hlaváč*

Contents

Fluorescence studies 2
Figure S1 2
Figure S2 3
Figure S3 4
Figure S4 5
Excitation and emission spectra 7
Figure S5 7
^{1}H and ^{13}C NMR spectra 12
Compound 1 (DMSO-d6) 12
Compound 2 (DMSO-d6) 13
Compound 3 (DMSO-d6) 14
Compound 4 (DMSO-d6) 15
Compound 5 (DMSO-d6) 16
Compound 6 (DMSO-d6) 17
Compound 7 (DMSO-d6) 18
Compound 8 (DMSO-d6) 19
Compound 9 (DMSO-d6) 20
Compound 10 (DMSO-d6) 21
Compound 11 (DMSO-d6) 22
Compound 12 (DMSO-d6) 23
Compound 13 (DMSO-d6) 24
Compound 14 (DMSO-d6) 25
Compound 15 (DMSO-d6) 26
Compound 18 ($CDCl_3$) 27

Fluorescence studies

Figure S1: Time monitored cleavage of conjugate **11** in medium without GSH (black), after treatment of the HeLa cells (red) or HeLa cells pretreated by 20 mM GSH (blue) with conjugate **11**. Fluorescence ratio I_{485}/I_{510} is reported as ratio of emission intensities at 530nm after excitation at 485nm and 510nm.

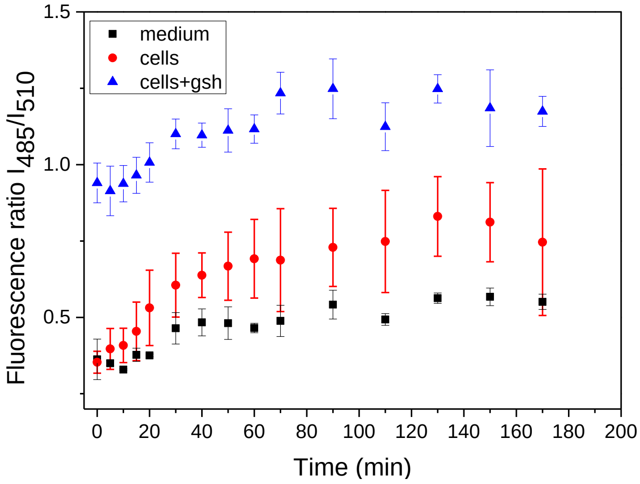

Figure S2: Time monitored cleavage of conjugate **12** in medium without GSH (black), after treatment of the HeLa cells (red) or HeLa cells pretreated by 20 mM GSH (blue) with conjugate **12**. Fluorescence ratio I_{485}/I_{510} is reported as ratio of emission intensities at 530nm after excitation at 485nm and 510nm.

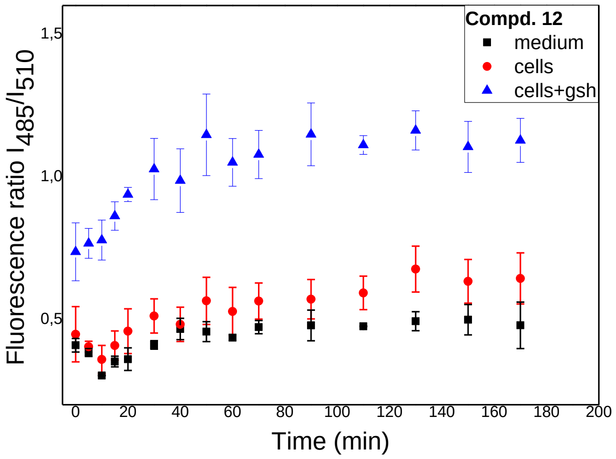

Figure S3: Time monitored cleavage of conjugate **14** in medium without GSH (black), after treatment of the HeLa cells (red) or HeLa cells pretreated by 20 mM GSH (blue) with conjugate **14**. Fluorescence ratio I_{485}/I_{510} is reported as ratio of emission intensities at 530nm after excitation at 485nm and 510nm.

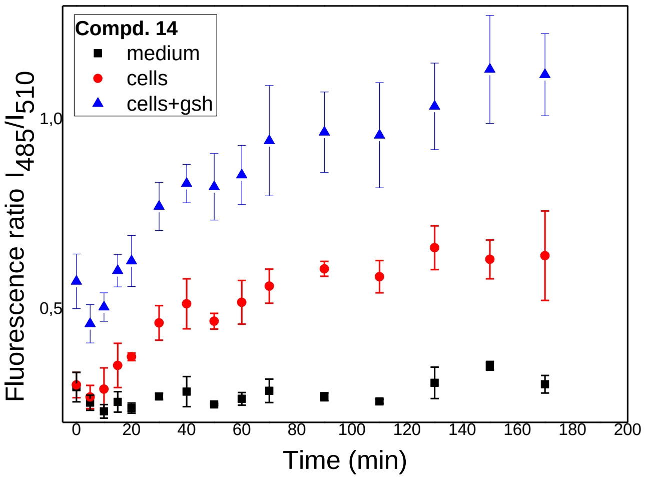

Figure S4: (A-E) Fluorescence ratio I485/I510 monitoring after treatment of non-cleavable conjugates **6-10** (5 µM) with GSH (5 mM) in DMSO/HEPES buffer 2:1 (0.1 M, pH 7.4, 37 °C). Fluorescence ratio I_{485}/I_{510} is reported as ratio of emission intensities at 530nm after excitation at 485nm and 510nm.

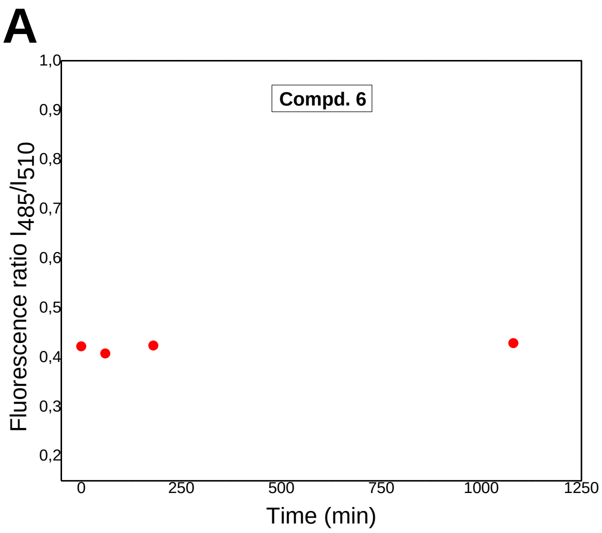

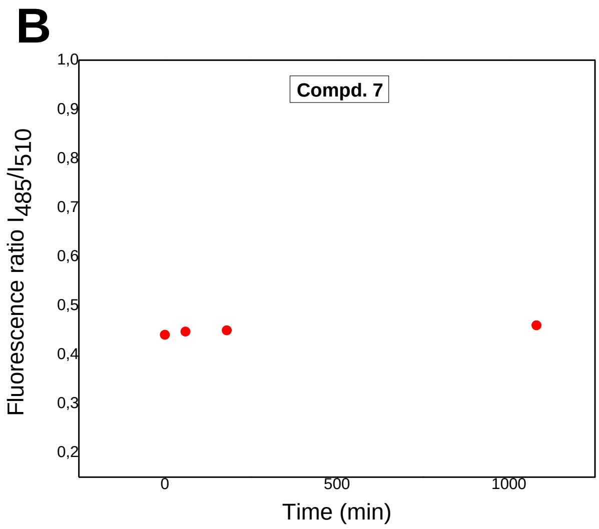

C

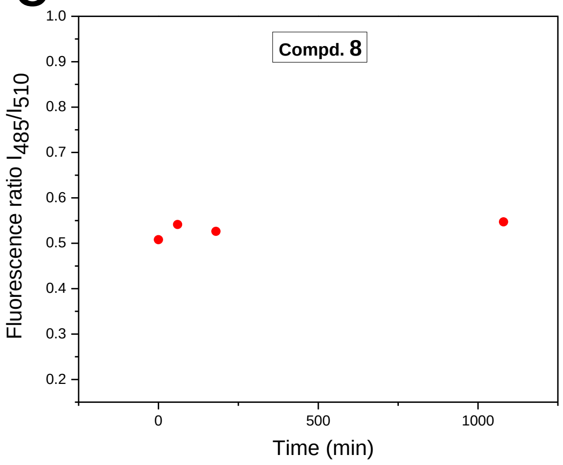
Compd. 8
Fluorescence ratio I_{485}/I_{510}
1.0
0.9
0.8
0.7
0.6
0.5
0.4
0.3
0.2
0
500
1000
Time (min)

D

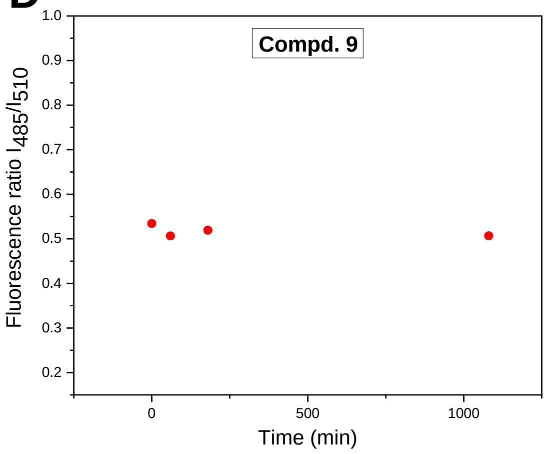
Compd. 9
Fluorescence ratio I_{485}/I_{510}
1.0
0.9
0.8
0.7
0.6
0.5
0.4
0.3
0.2
0
500
1000
Time (min)

E

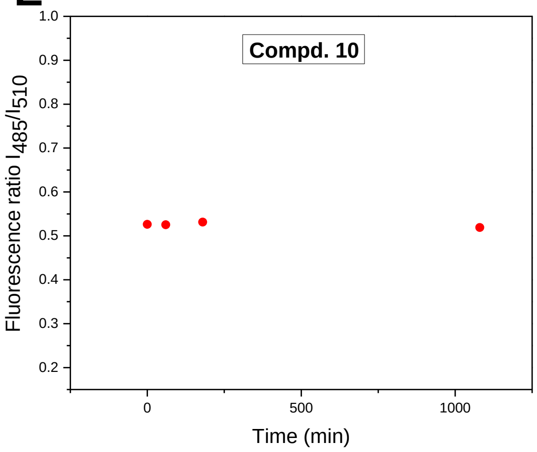
Compd. 10
Fluorescence ratio I_{485}/I_{510}
1.0
0.9
0.8
0.7
0.6
0.5
0.4
0.3
0.2
0
500
1000
Time (min)

Excitation and emission spectra

Figure S5: Normalized excitation (black line) and emission (red line) spectra of compounds **6-15**. All measurements were performed in DMSO/HEPES 2:1 with excitation wavelength λ_{exc} = 510 nm and source\detector slits width 2.5

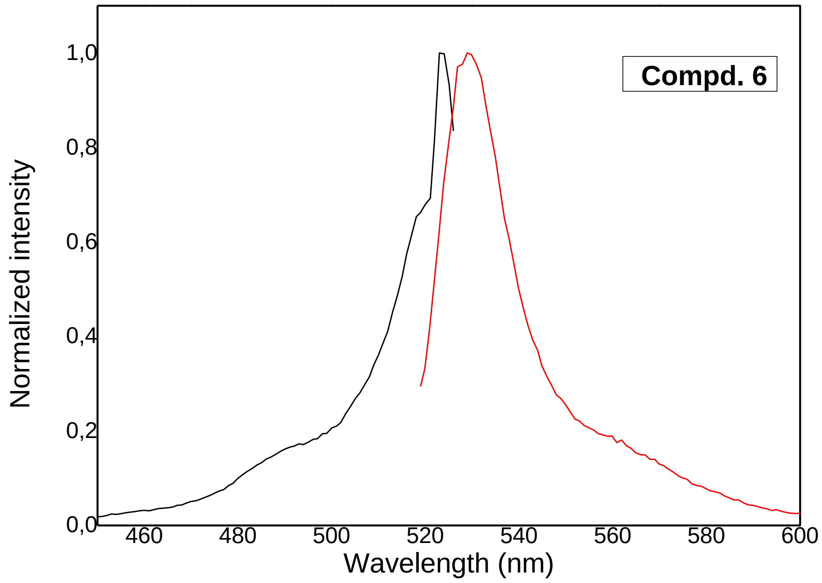

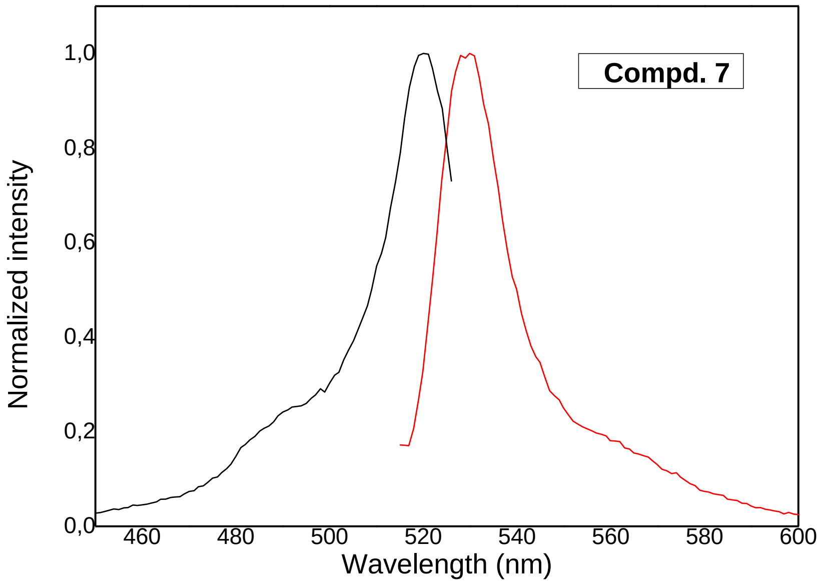

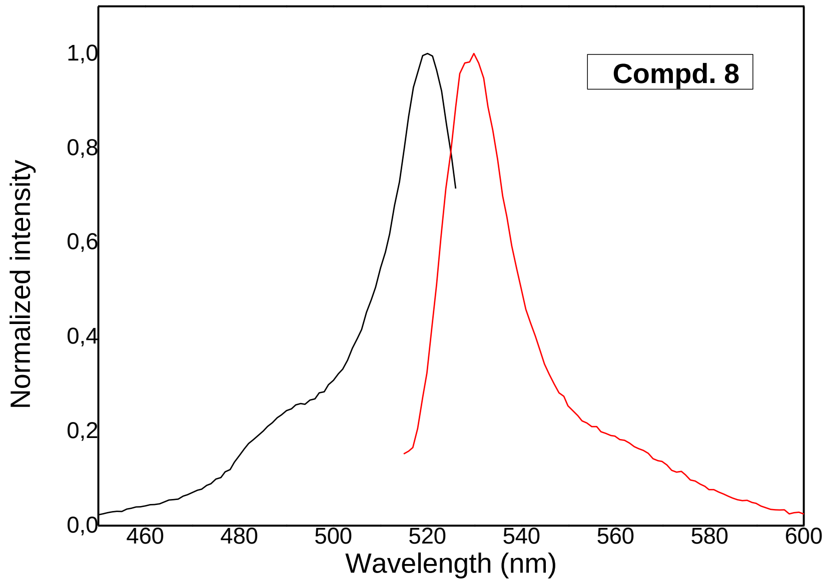
Compd. 8
Normalized intensity
1,0
0,8
0,6
0,4
0,2
0,0
460
480
500
520
540
560
580
600
Wavelength (nm)

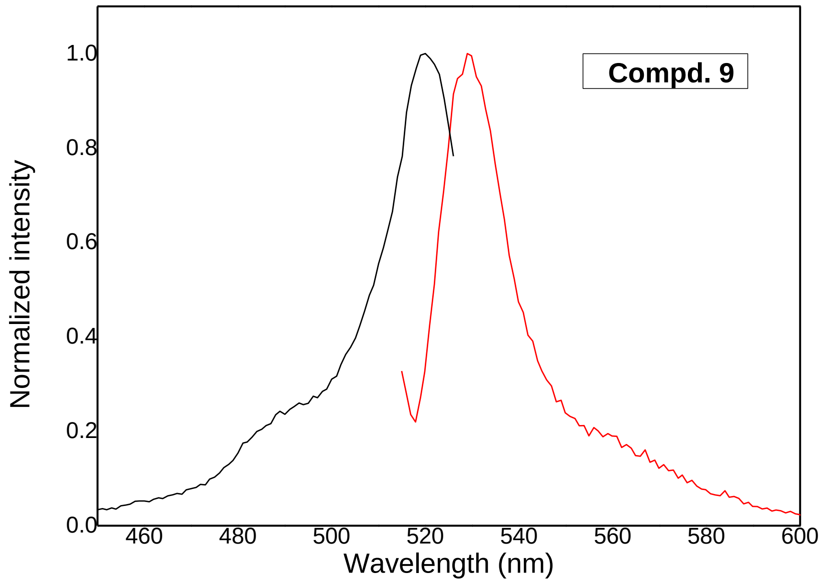
Compd. 9
Normalized intensity
1.0
0.8
0.6
0.4
0.2
0.0
460
480
500
520
540
560
580
600
Wavelength (nm)

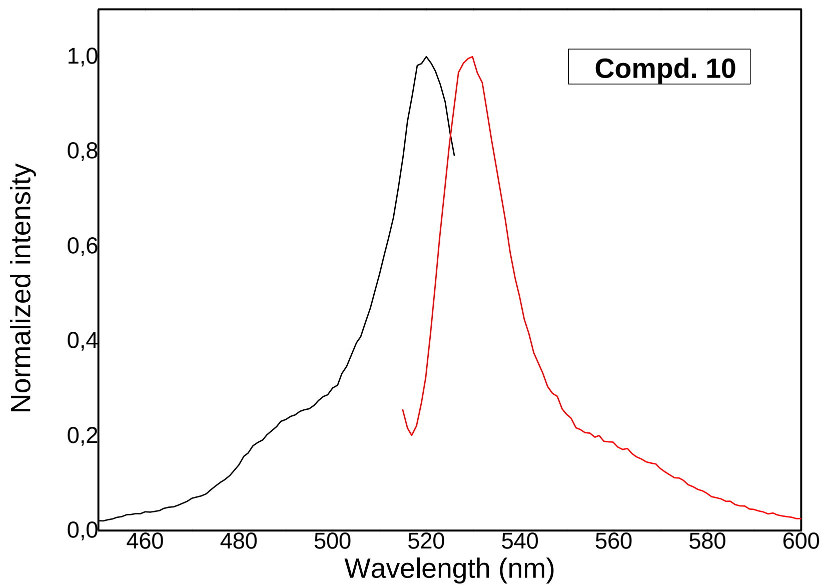
Compd. 10
Normalized intensity
1,0
0,8
0,6
0,4
0,2
0,0
460
480
500
520
540
560
580
600
Wavelength (nm)

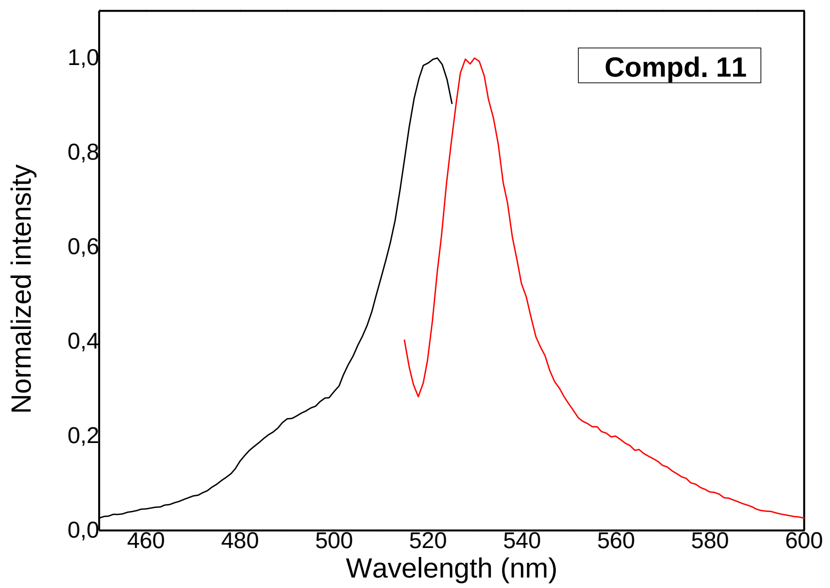
Compd. 11
Normalized intensity
1,0
0,8
0,6
0,4
0,2
0,0
460
480
500
520
540
560
580
600
Wavelength (nm)

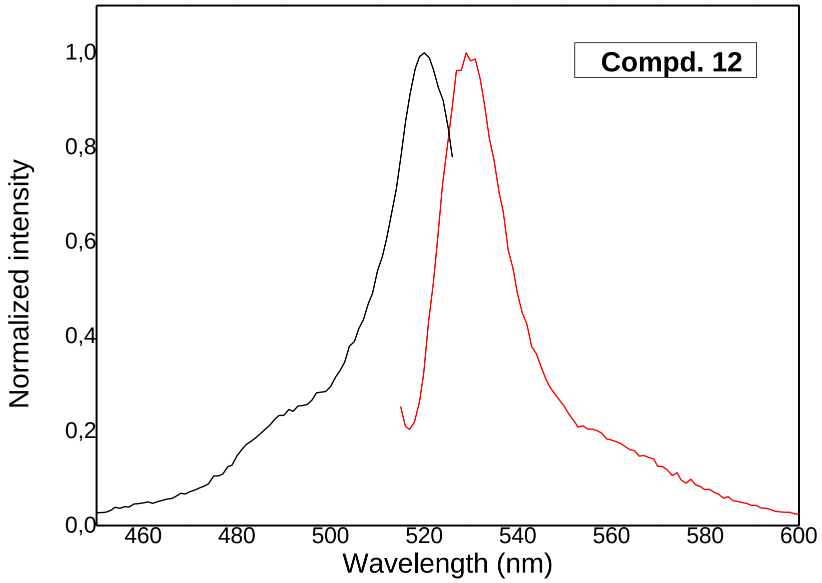
Compd. 12
Normalized intensity
1,0
0,8
0,6
0,4
0,2
0,0
460
480
500
520
540
560
580
600
Wavelength (nm)

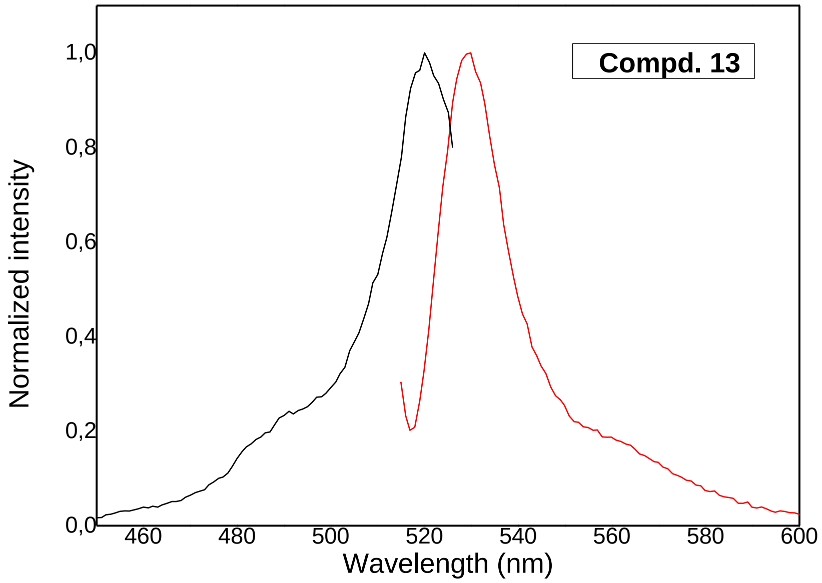
Compd. 13
Normalized intensity
1,0
0,8
0,6
0,4
0,2
0,0
460
480
500
520
540
560
580
600
Wavelength (nm)

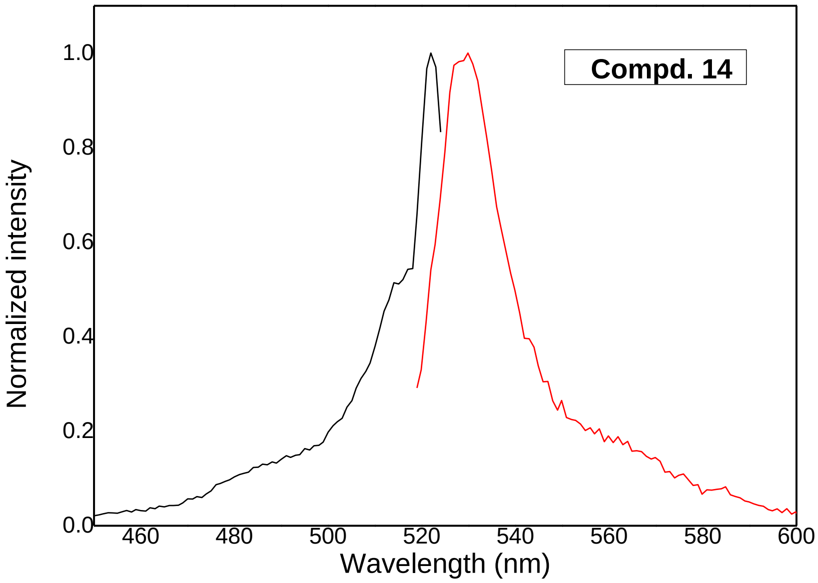
Compd. 14
Normalized intensity
Wavelength (nm)
1.0
0.8
0.6
0.4
0.2
0.0
460
480
500
520
540
560
580
600

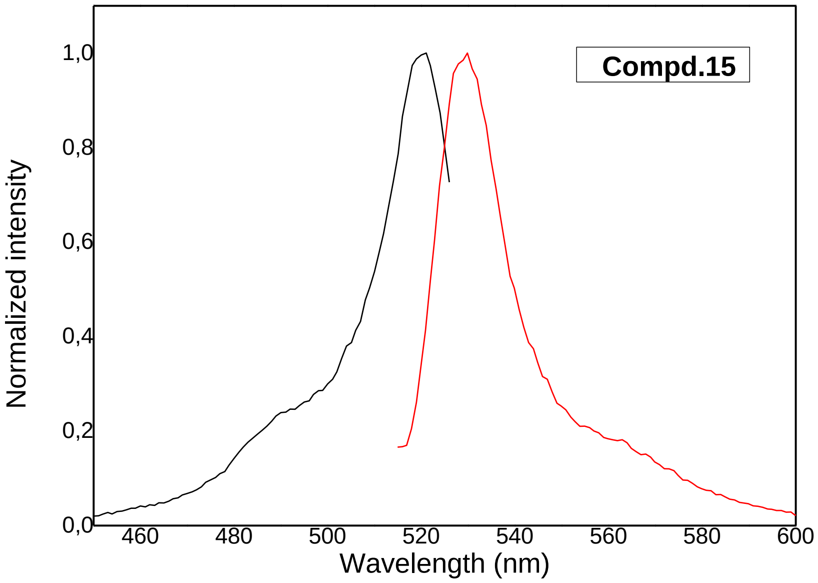
Compd.15
Normalized intensity
Wavelength (nm)
1,0
0,8
0,6
0,4
0,2
0,0
460
480
500
520
540
560
580
600

^{1}H and ^{13}C NMR spectra

Compound 1 (DMSO-d6)

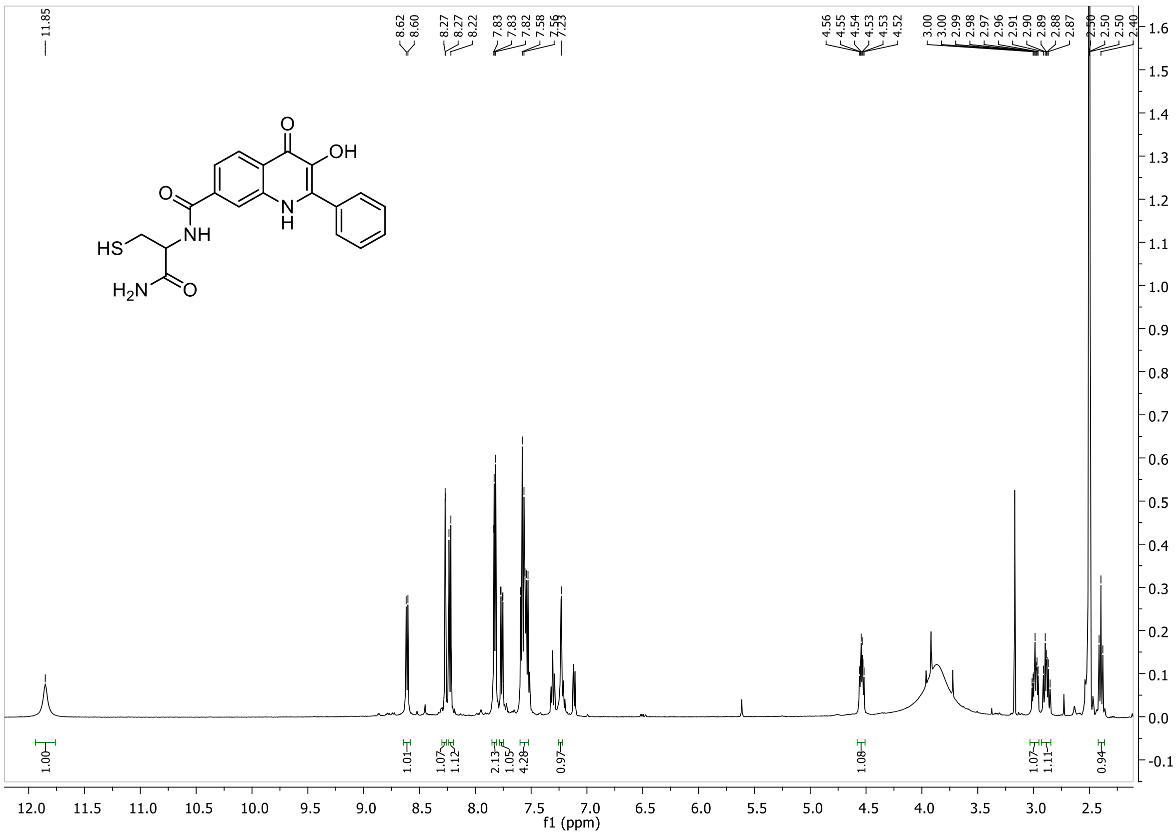

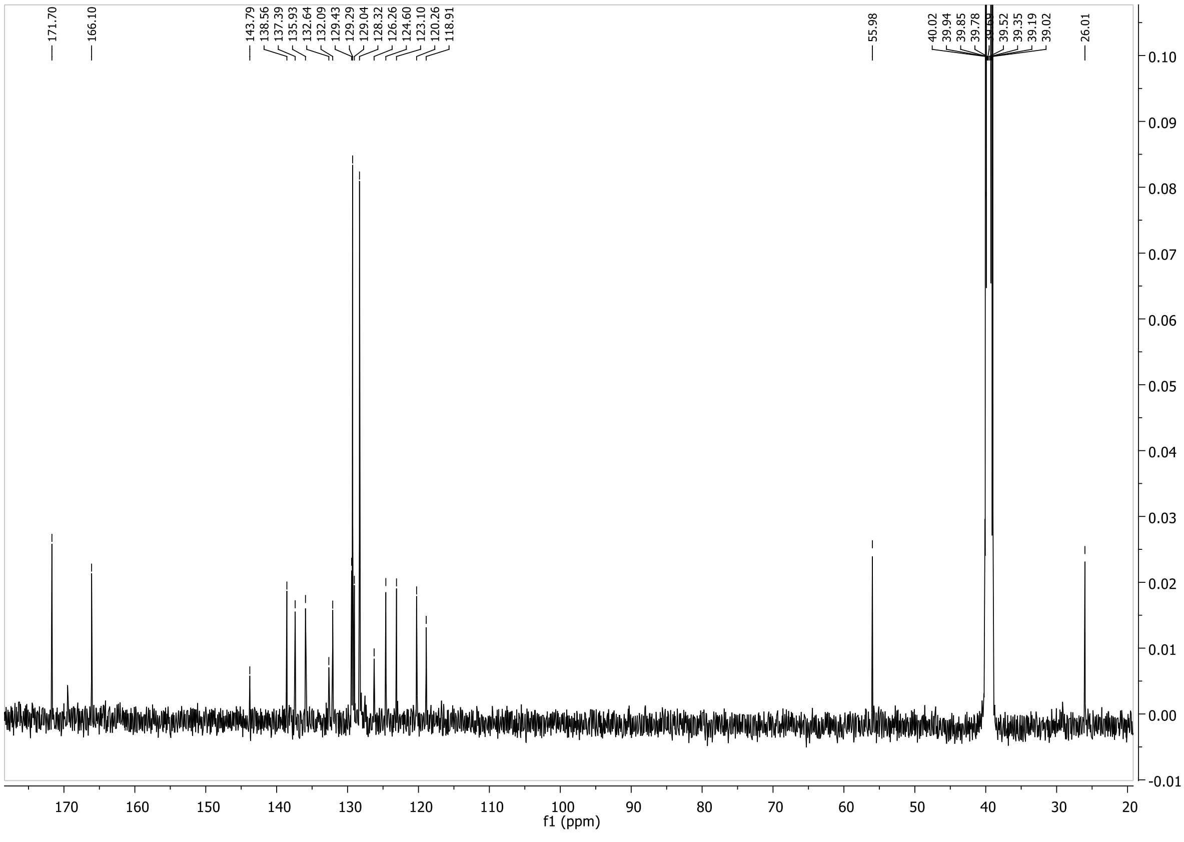

Compound 2 (DMSO-d6)

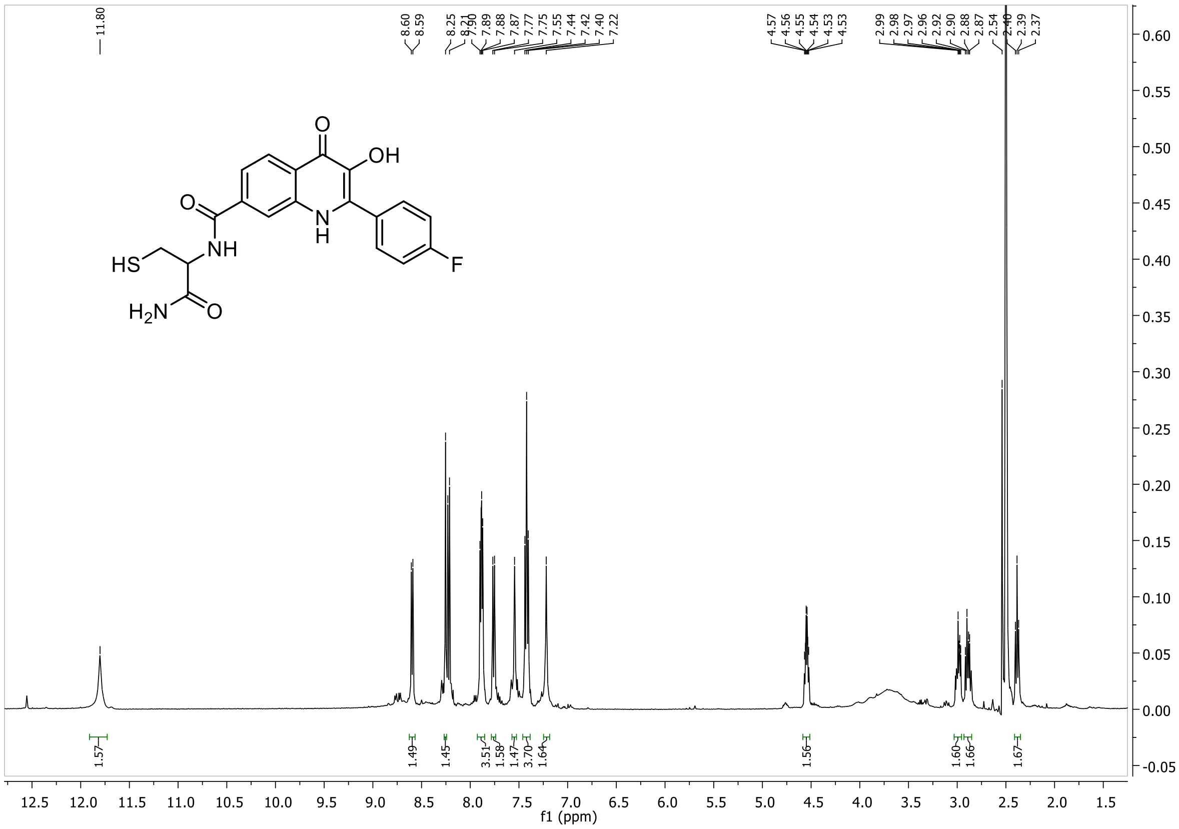

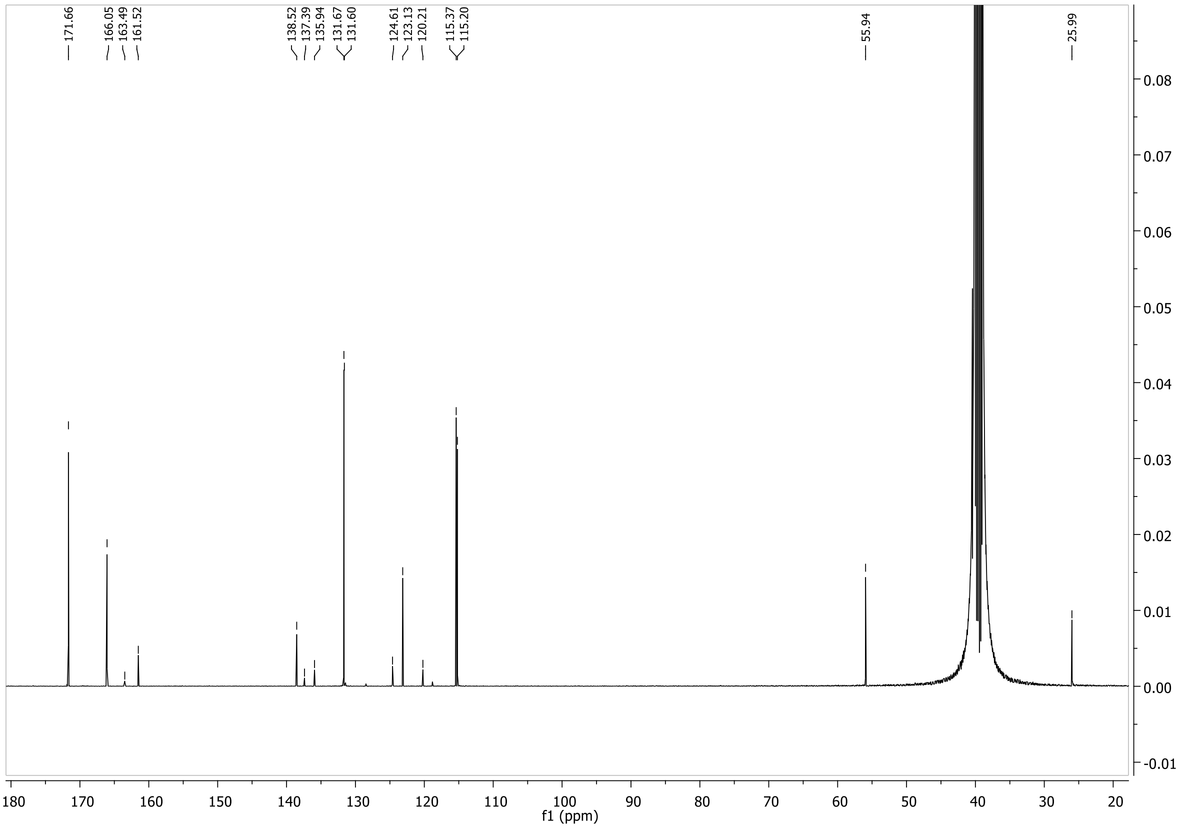

Compound 3 (DMSO-d6)

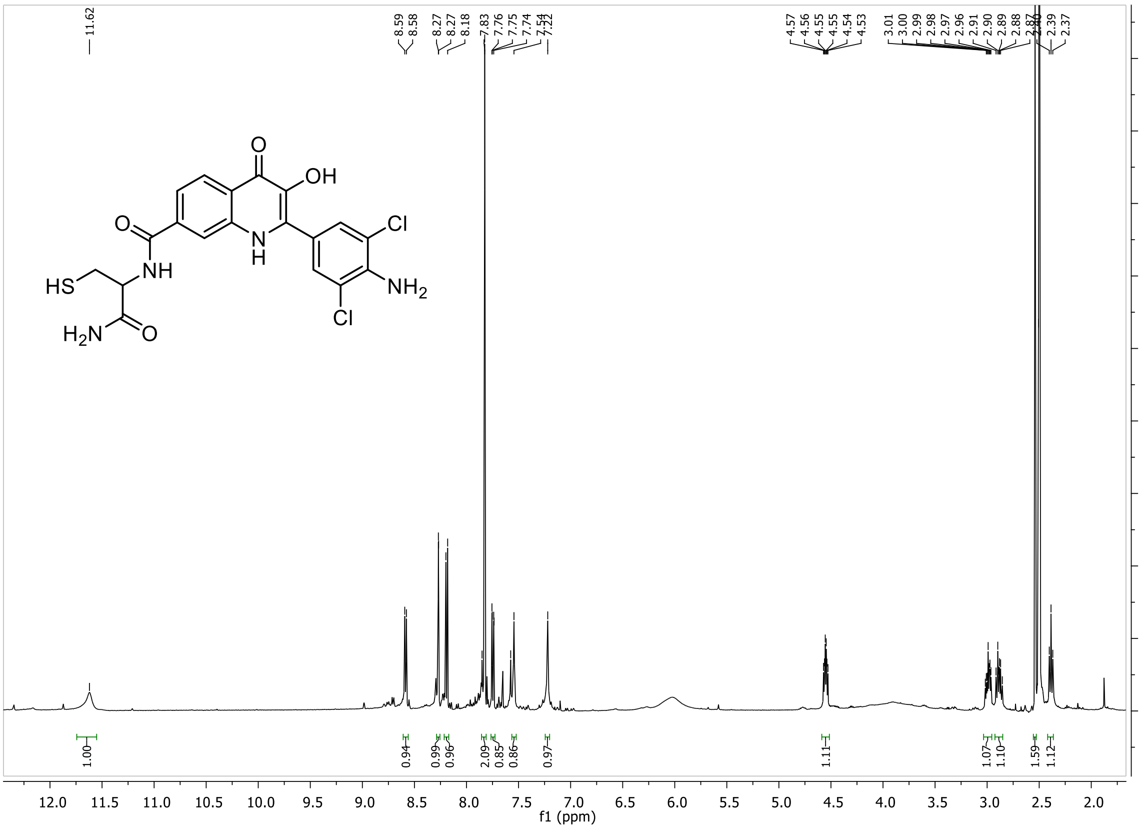

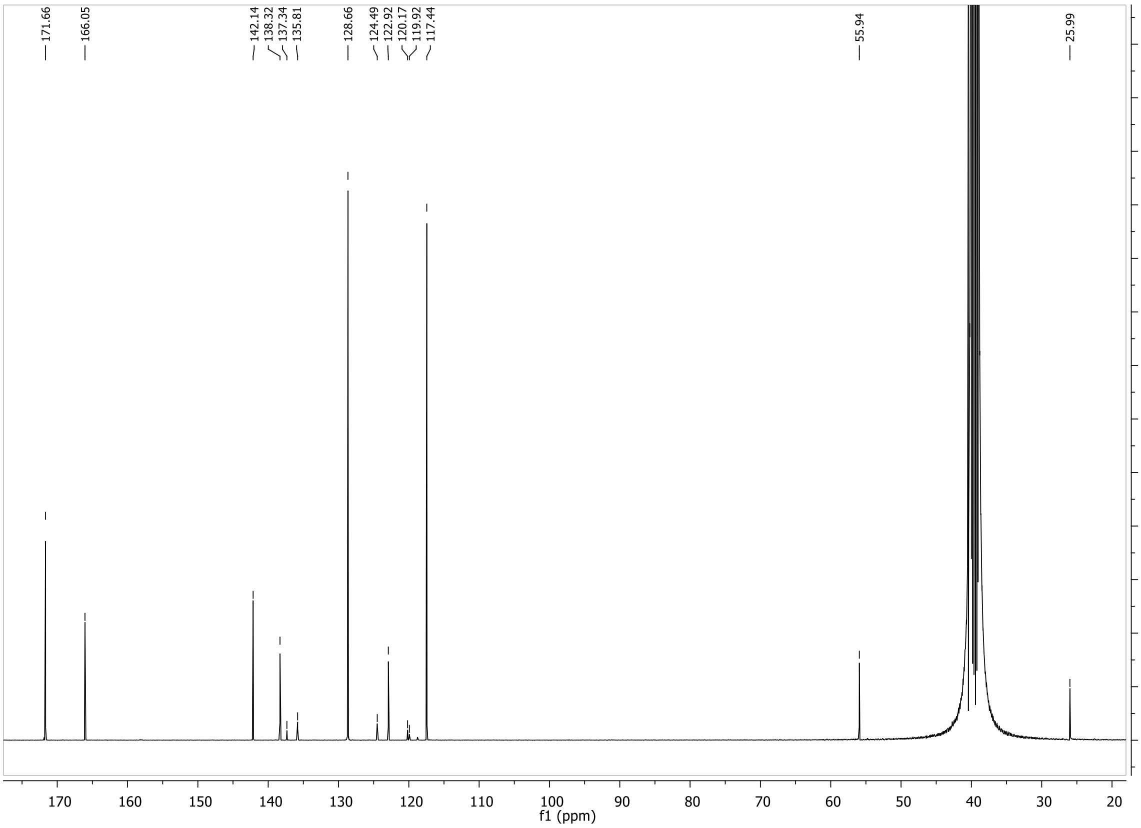

Compound 4 (DMSO-d6)

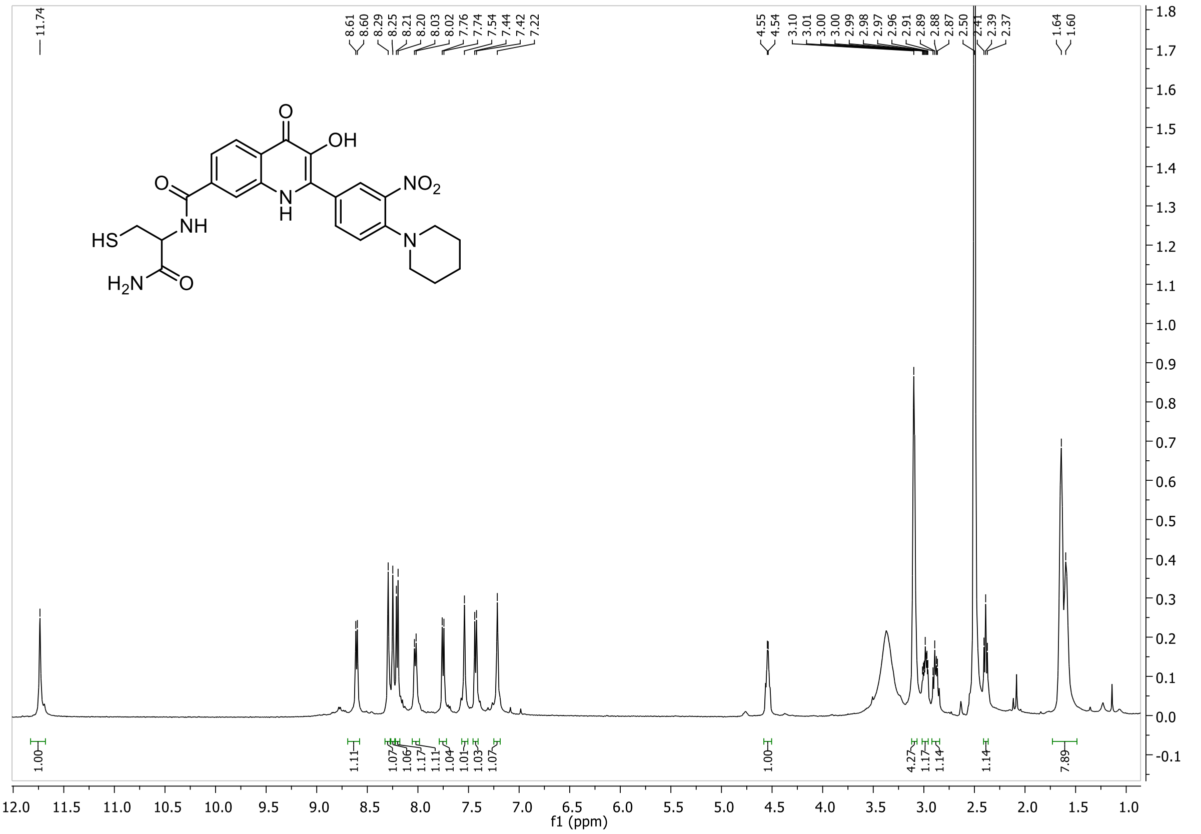

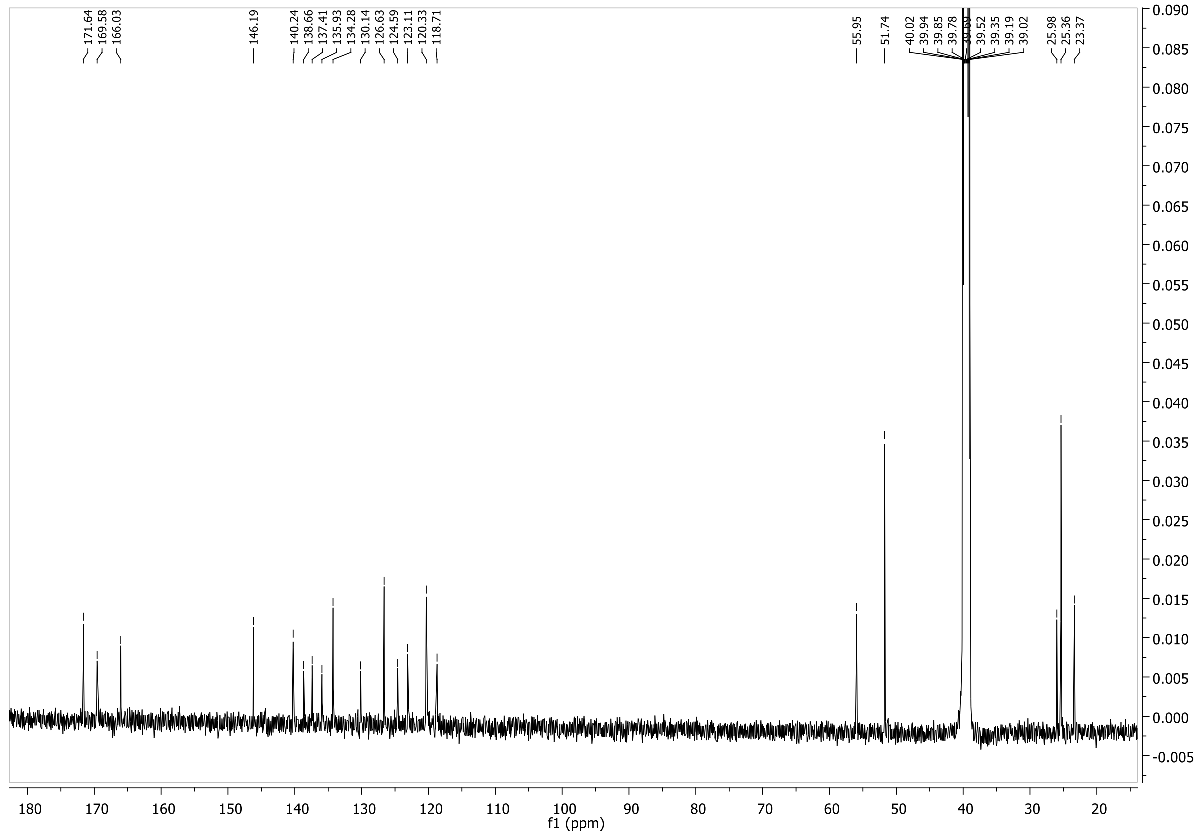

Compound 5 (DMSO-d6)

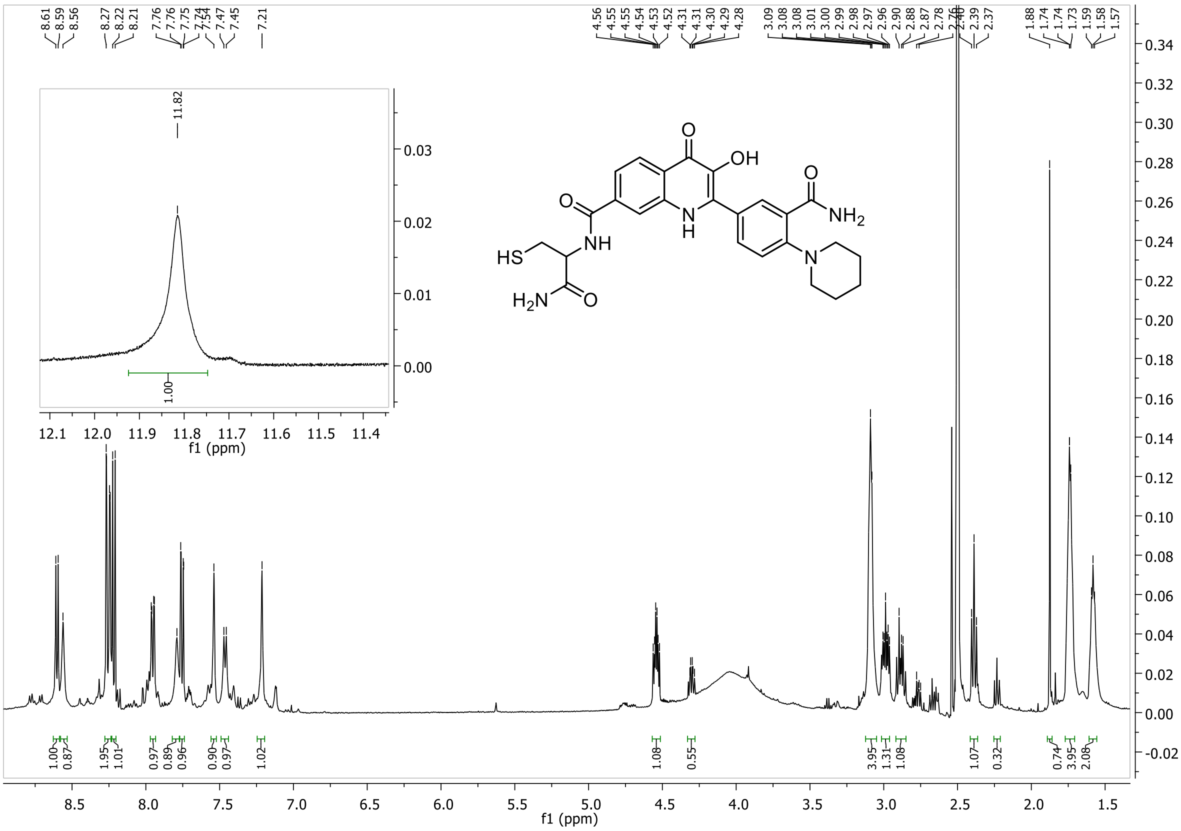

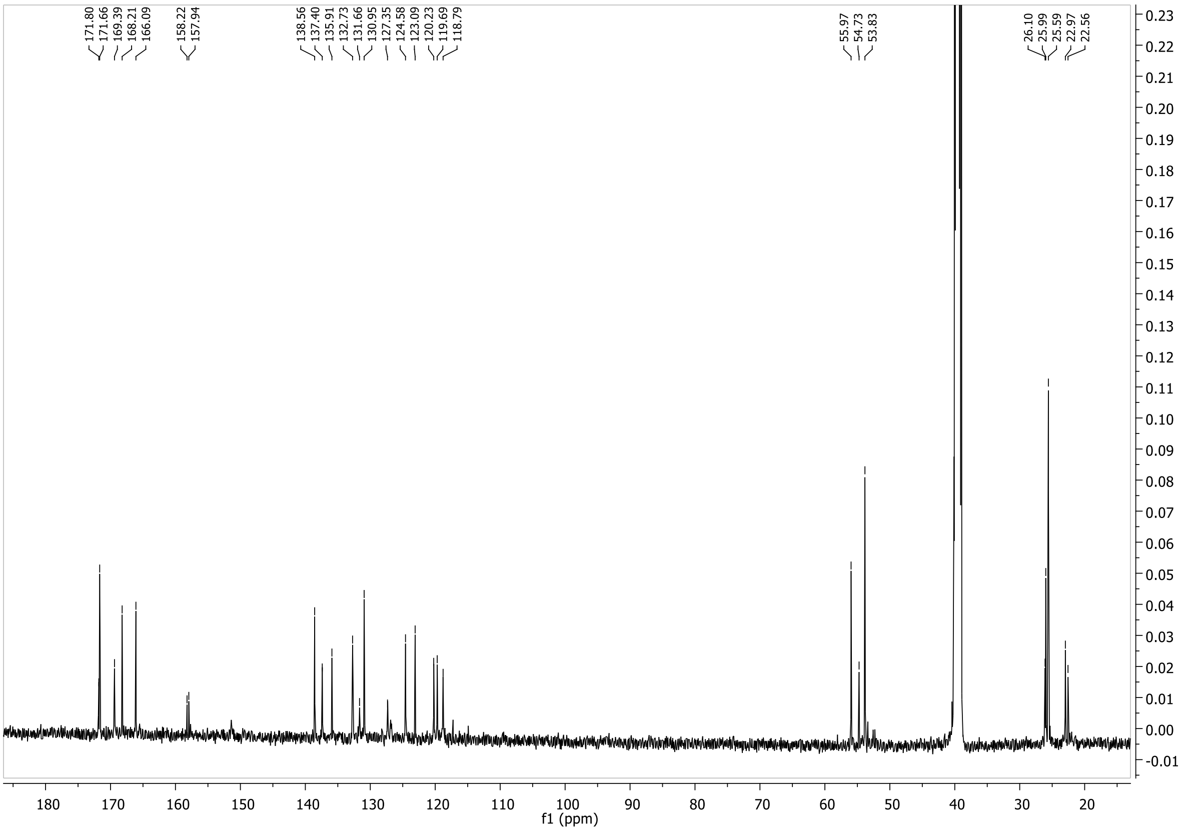

Compound 6 (DMSO-d6)

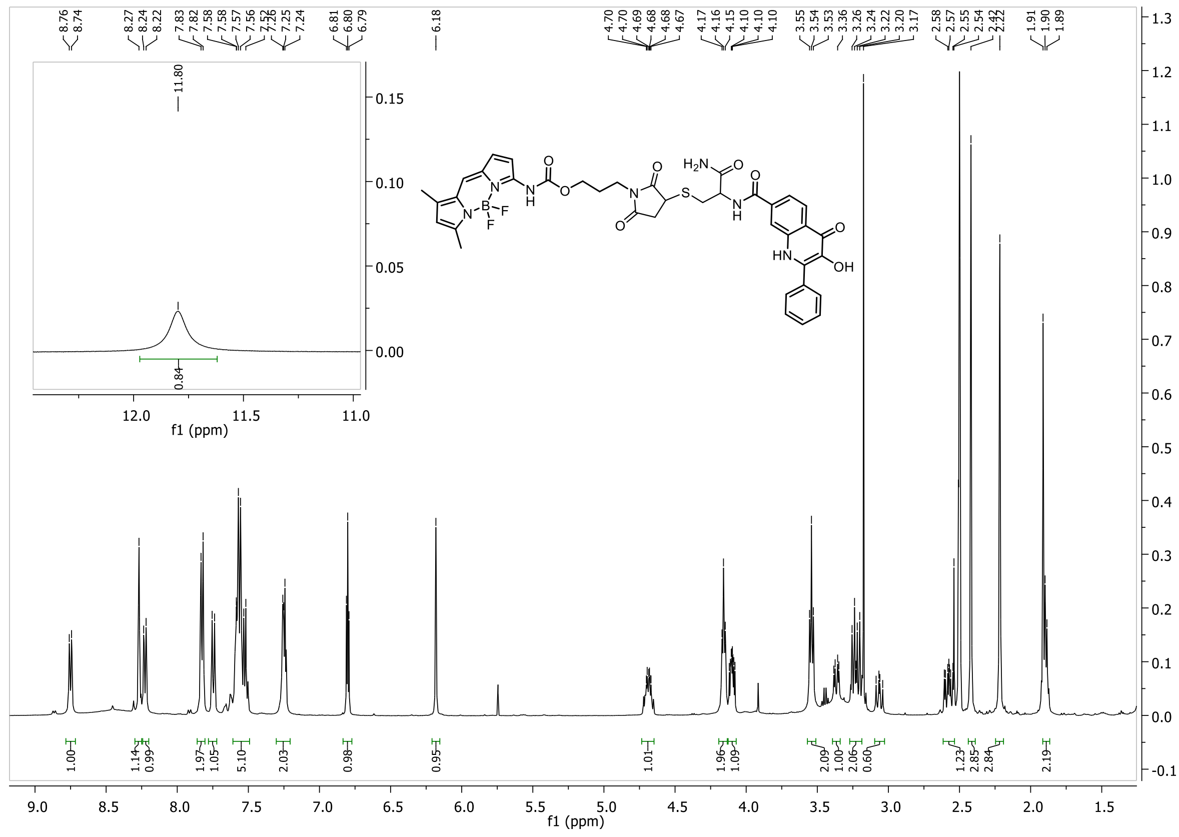

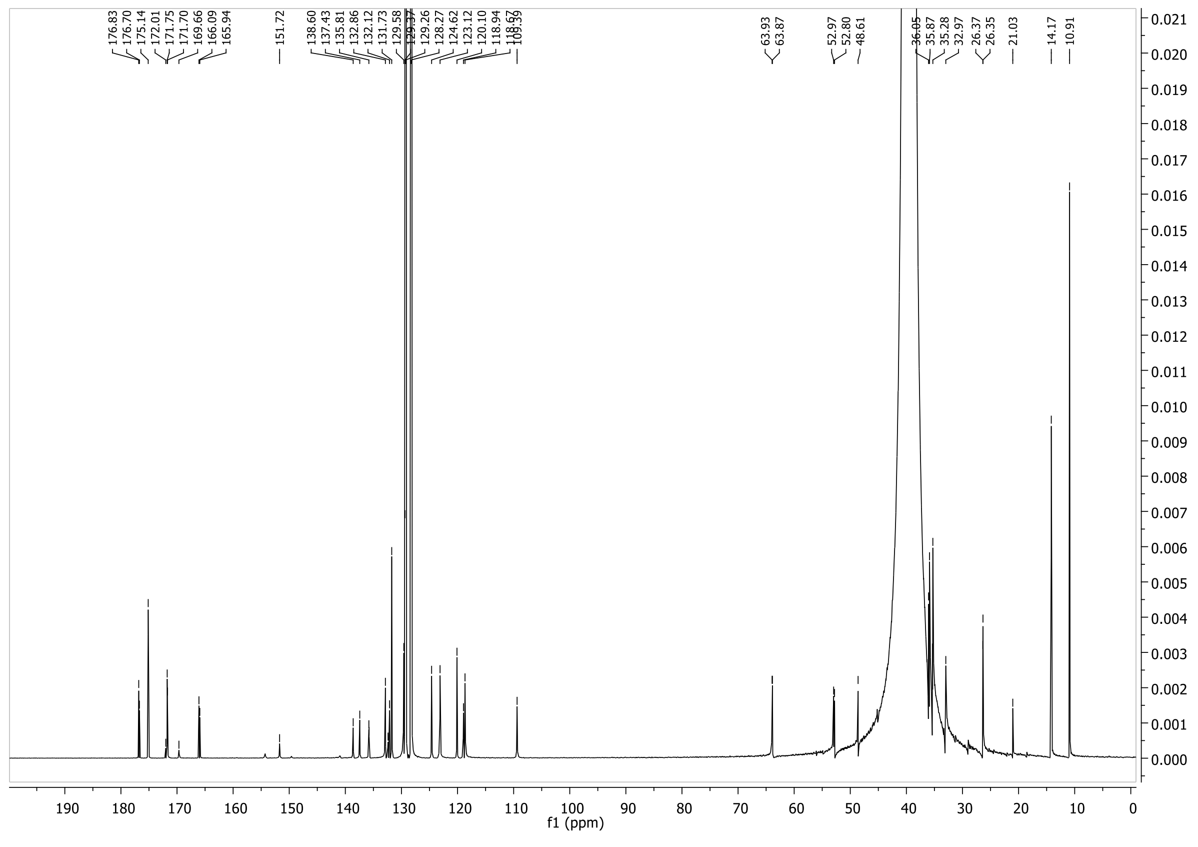

Compound 7 (DMSO-d6)

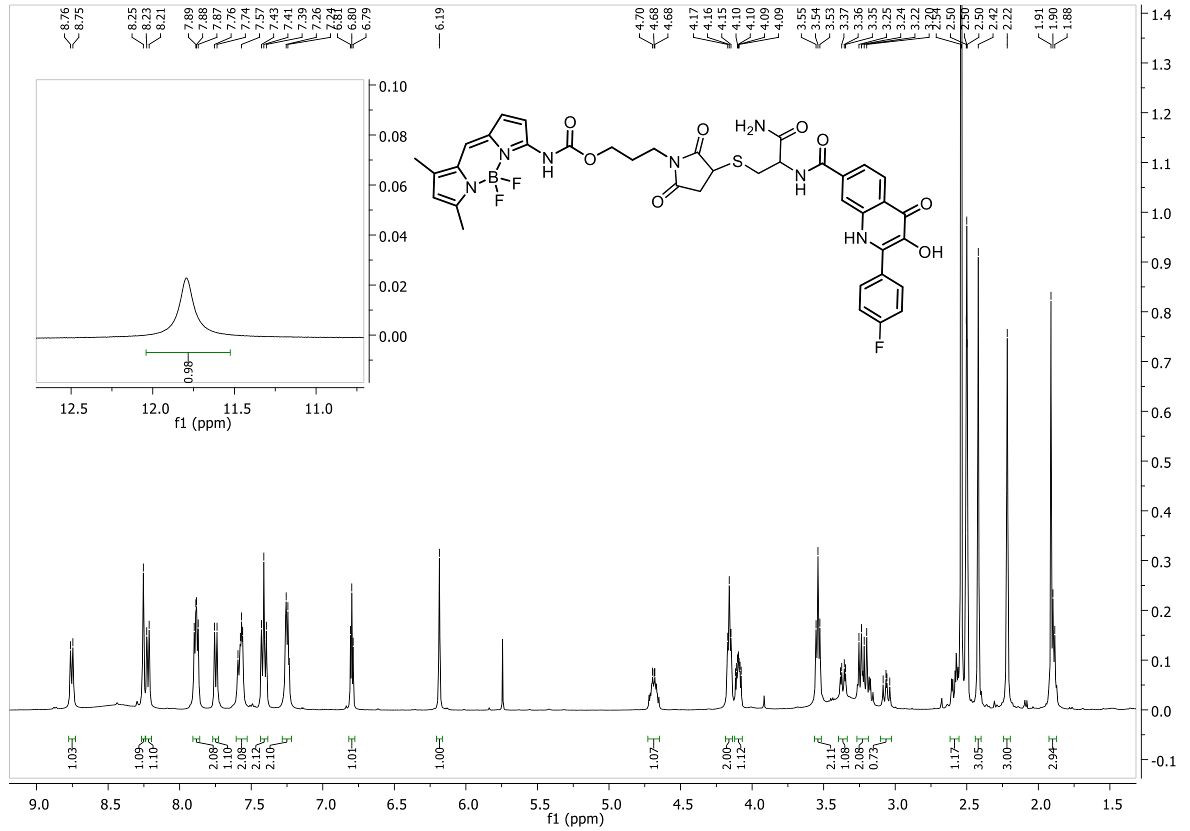

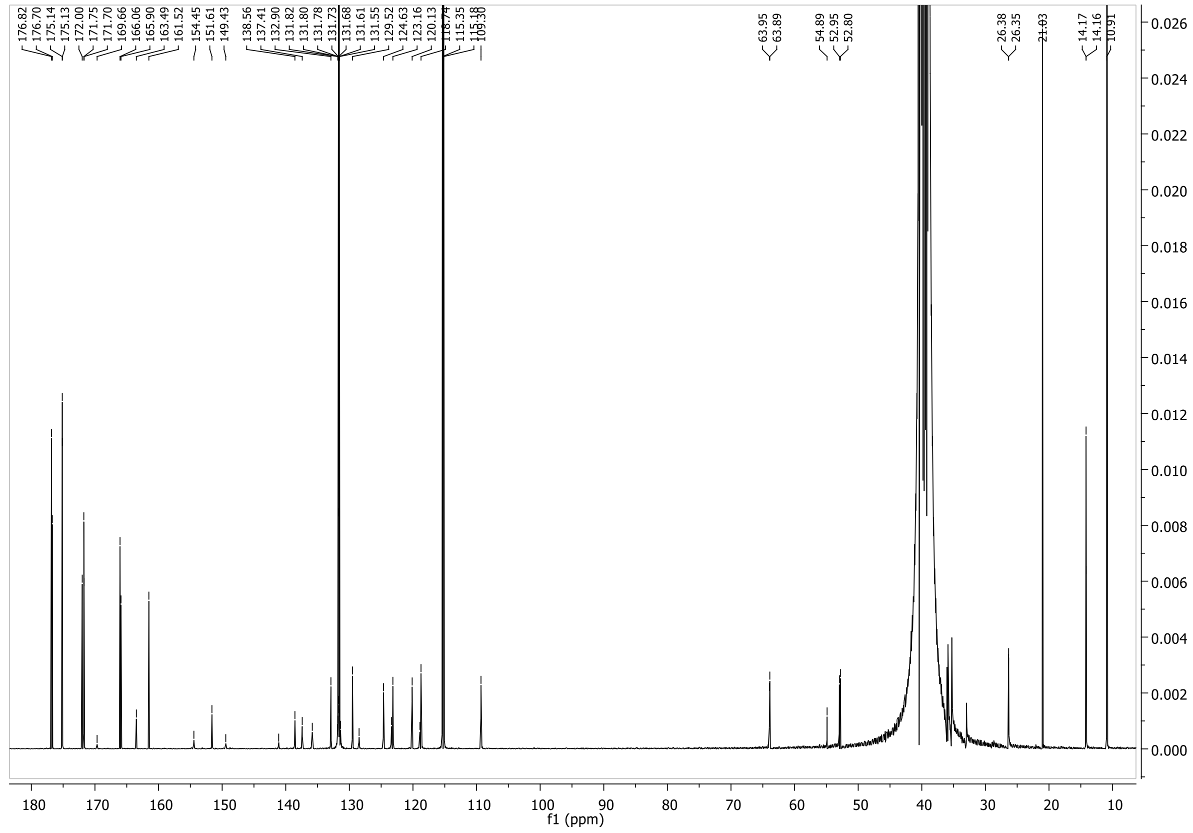

Compound 8 (DMSO-d6)

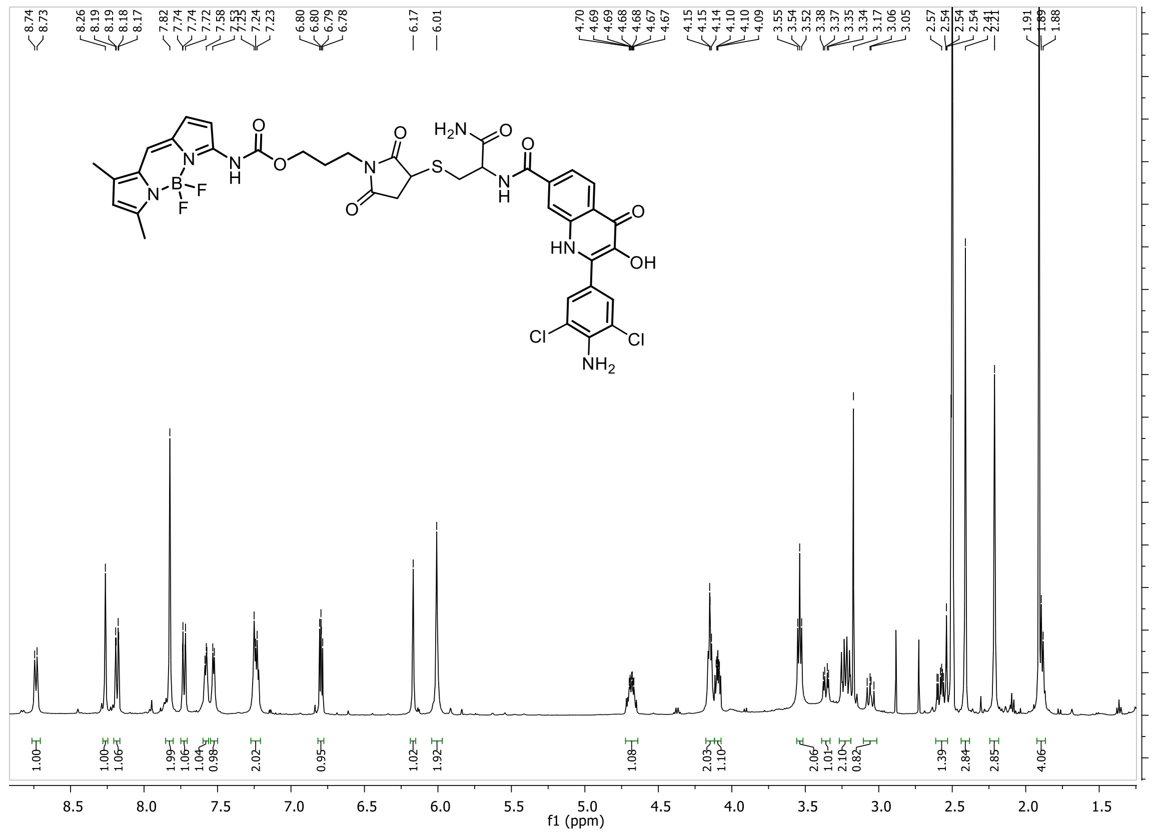

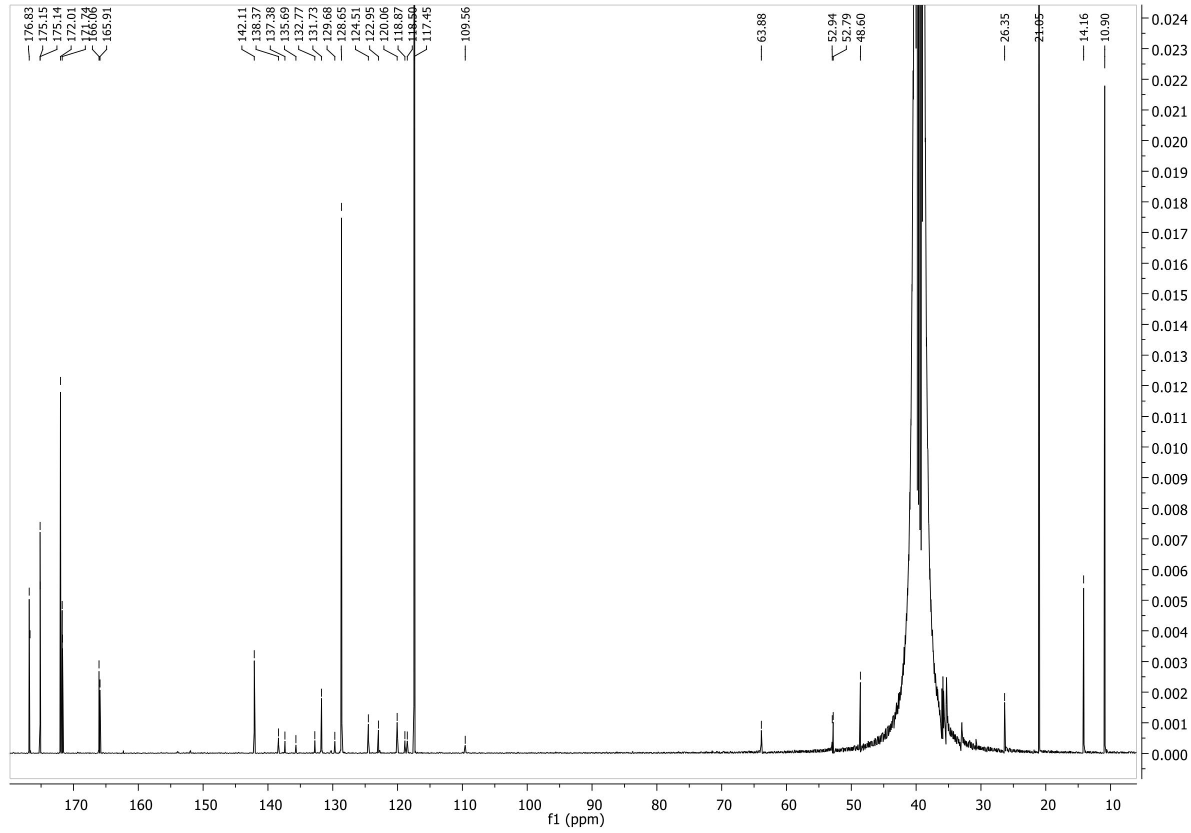

Compound 9 (DMSO-d6)

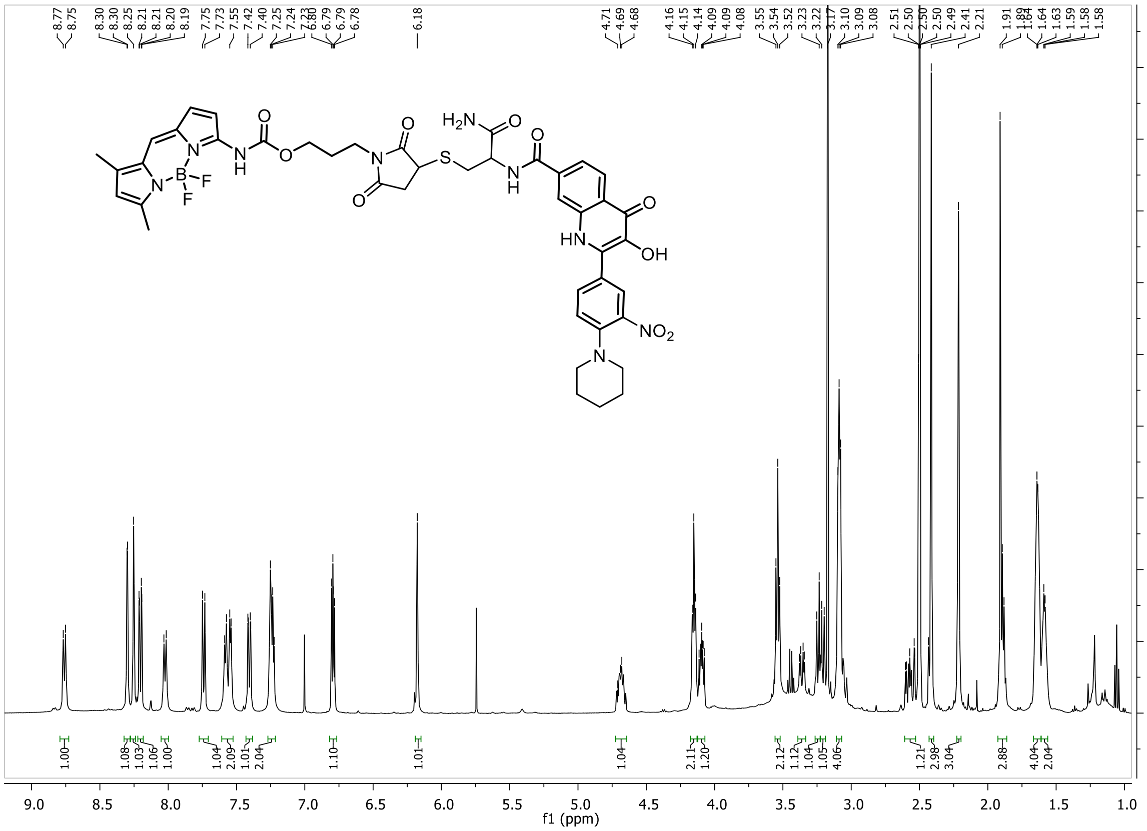

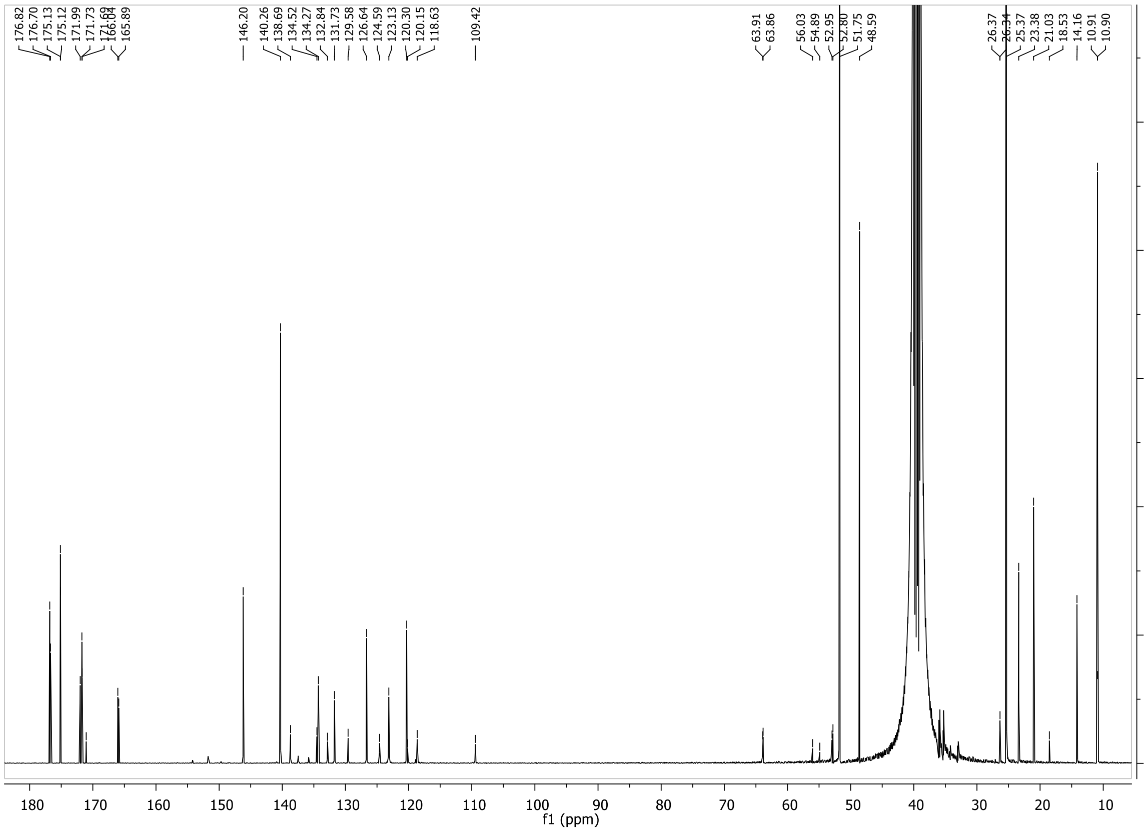

Compound 10 (DMSO-d6)

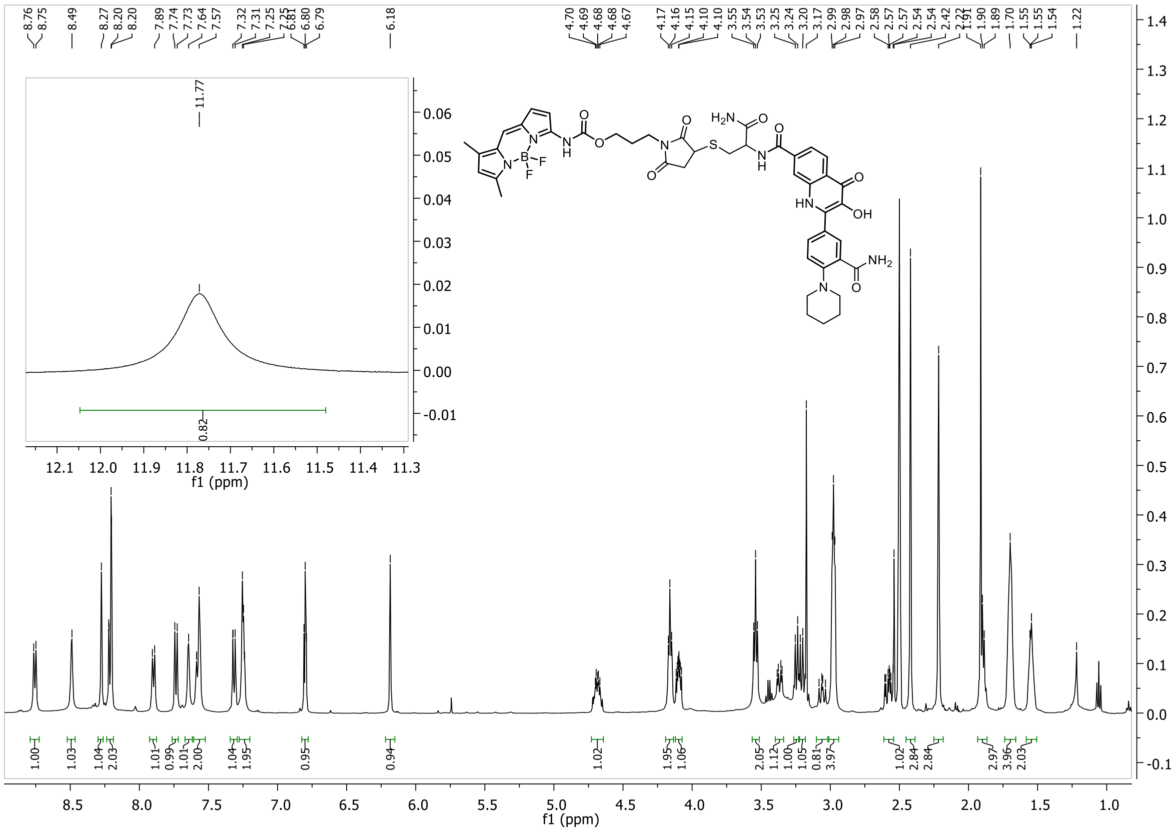

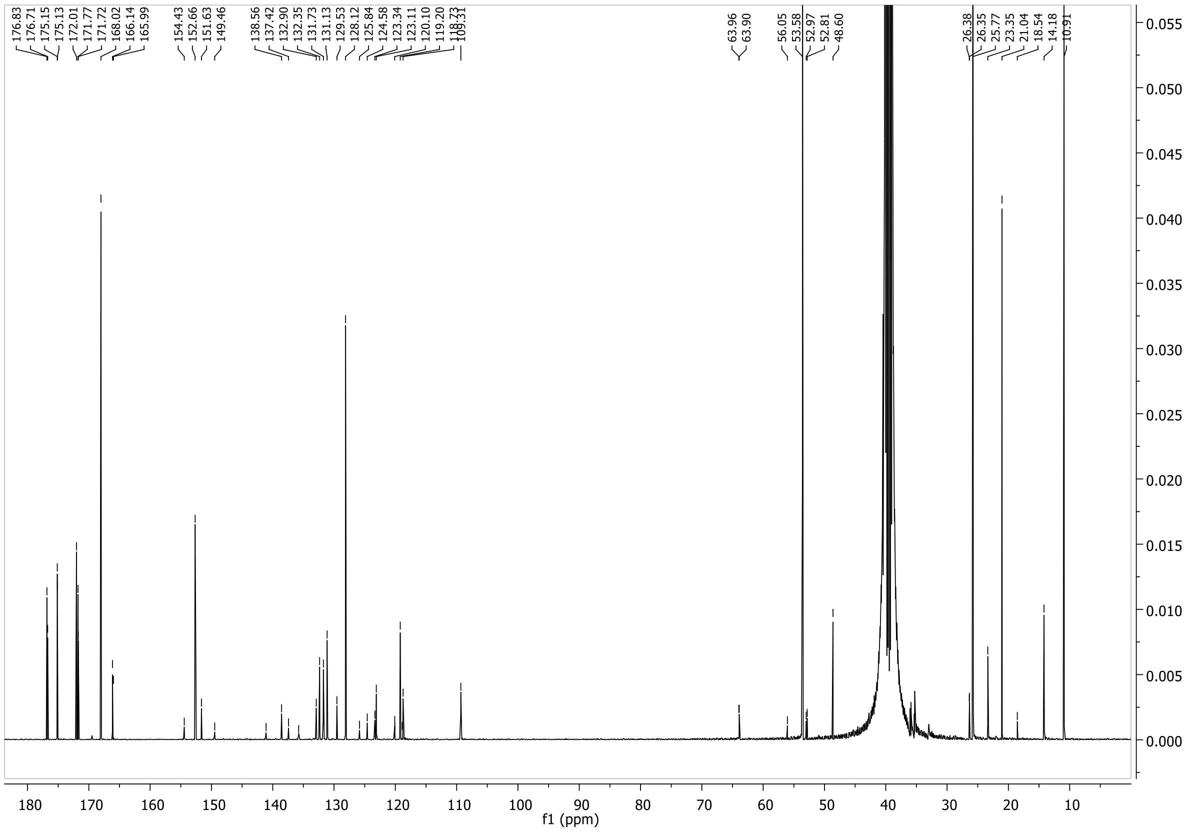

Compound 11 (DMSO-d6)

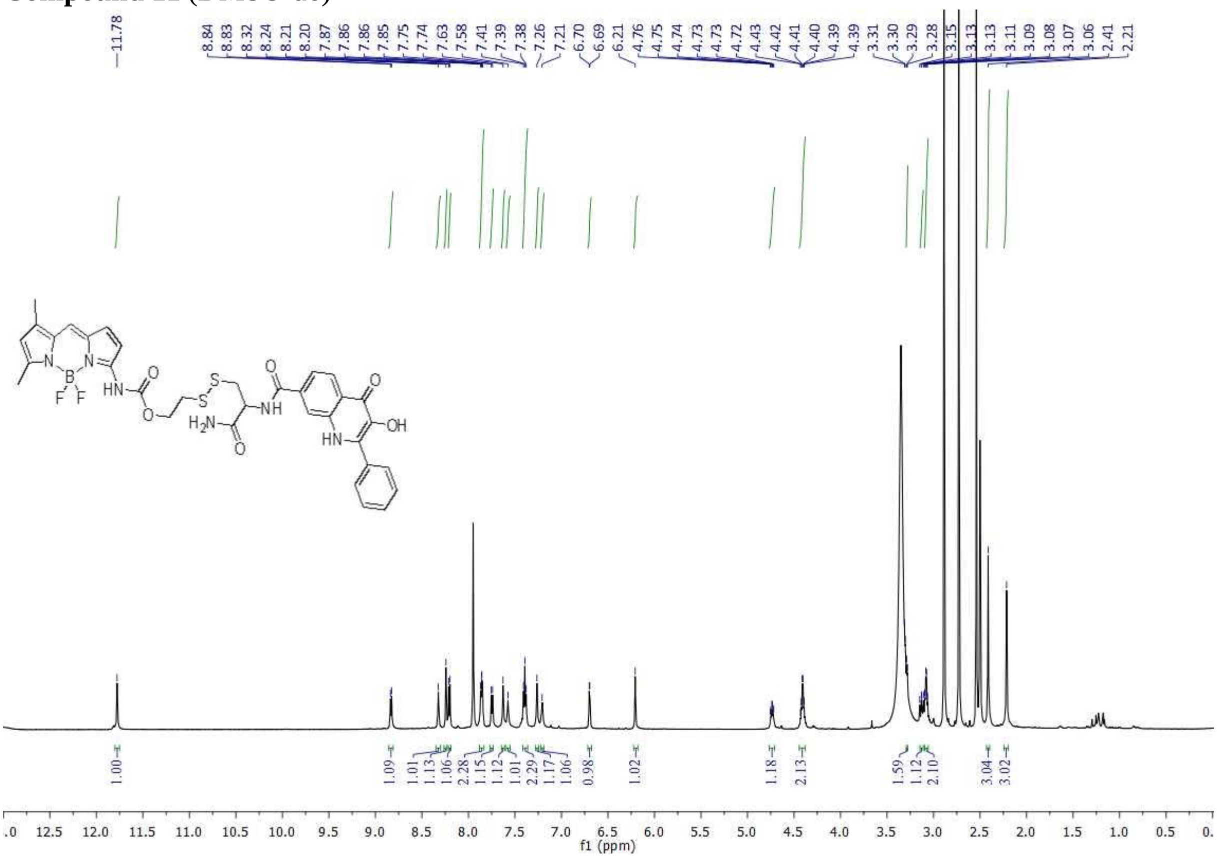

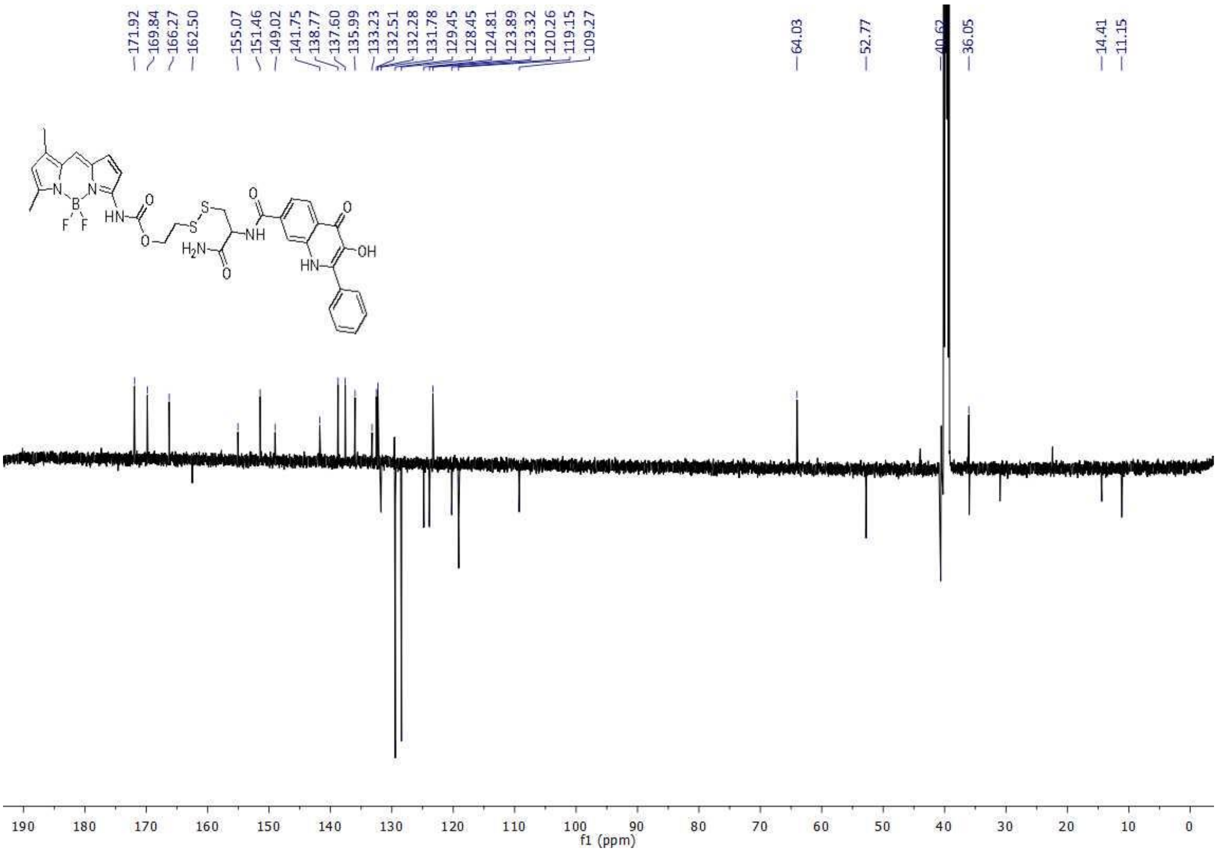

Compound 12 (DMSO-d6)

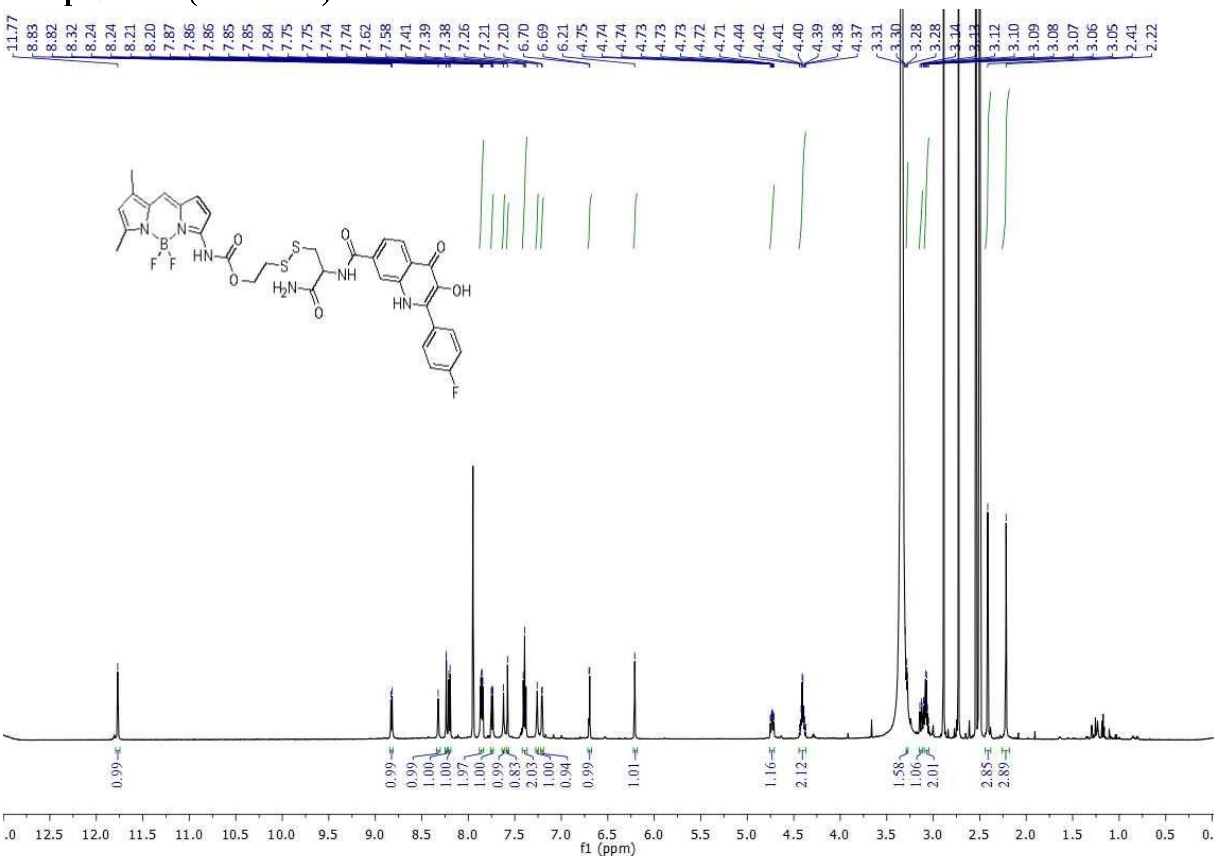

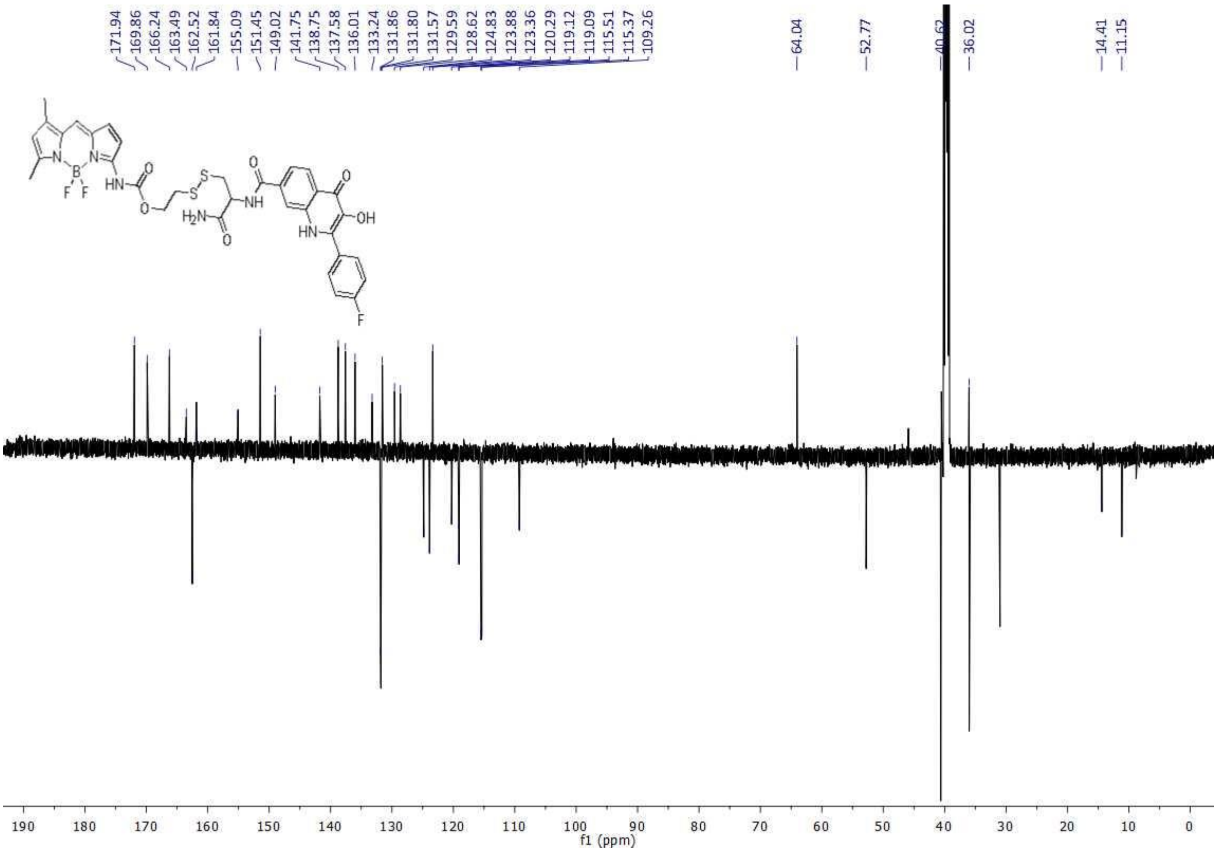

Compound 13 (DMSO-d6)

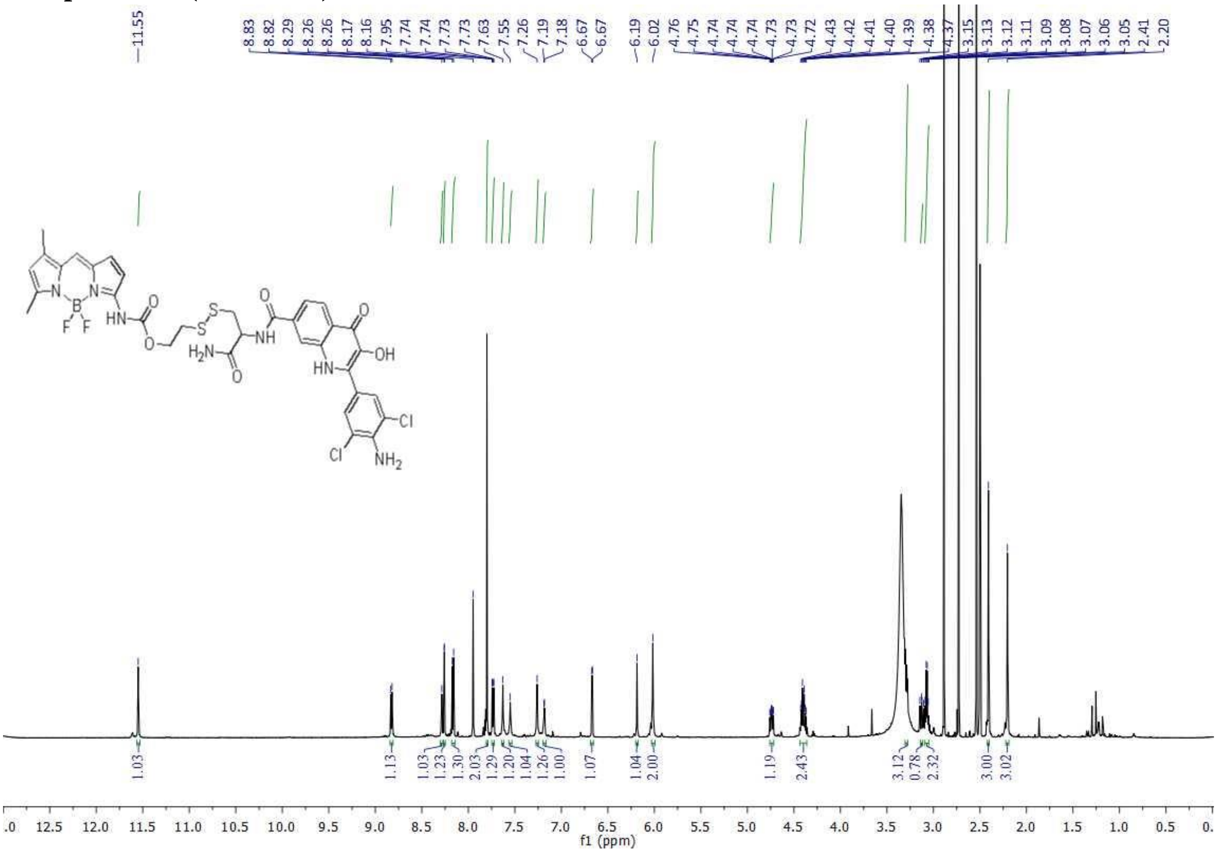

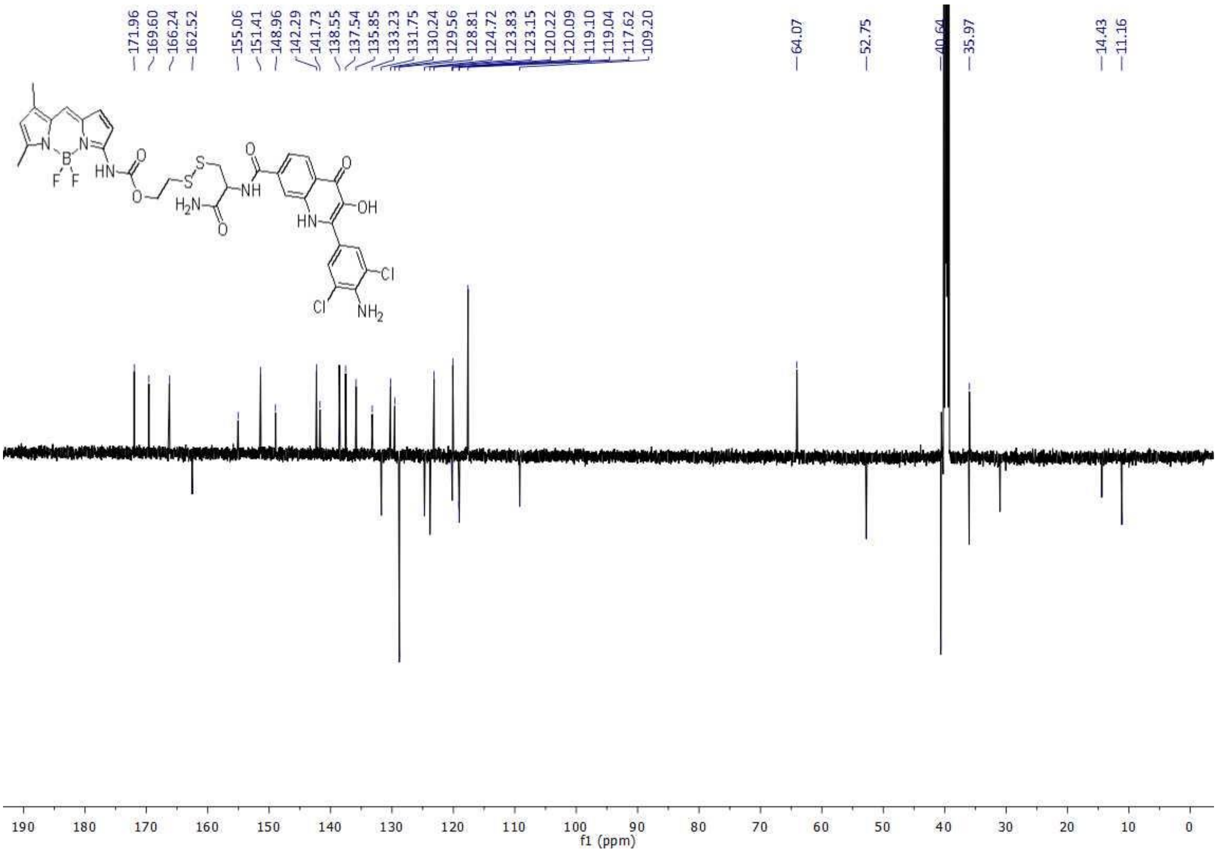

Compound 14 (DMSO-d6)

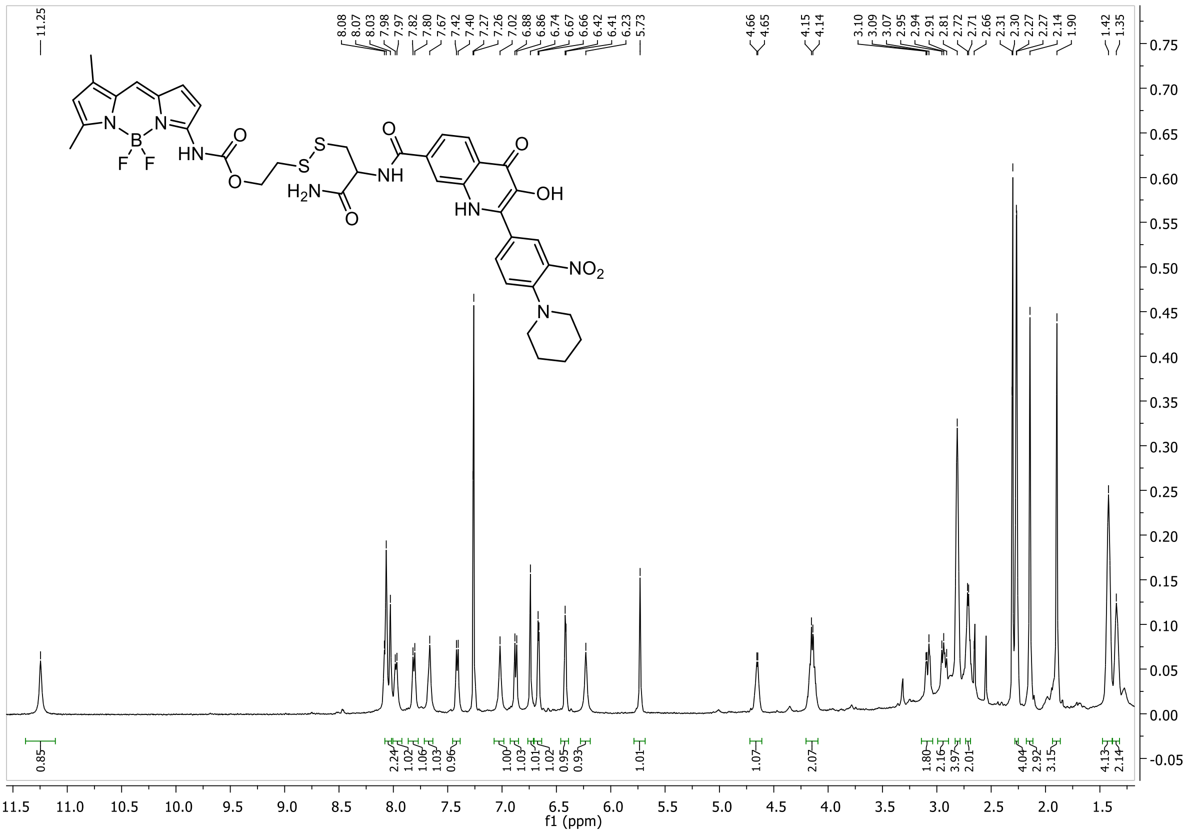

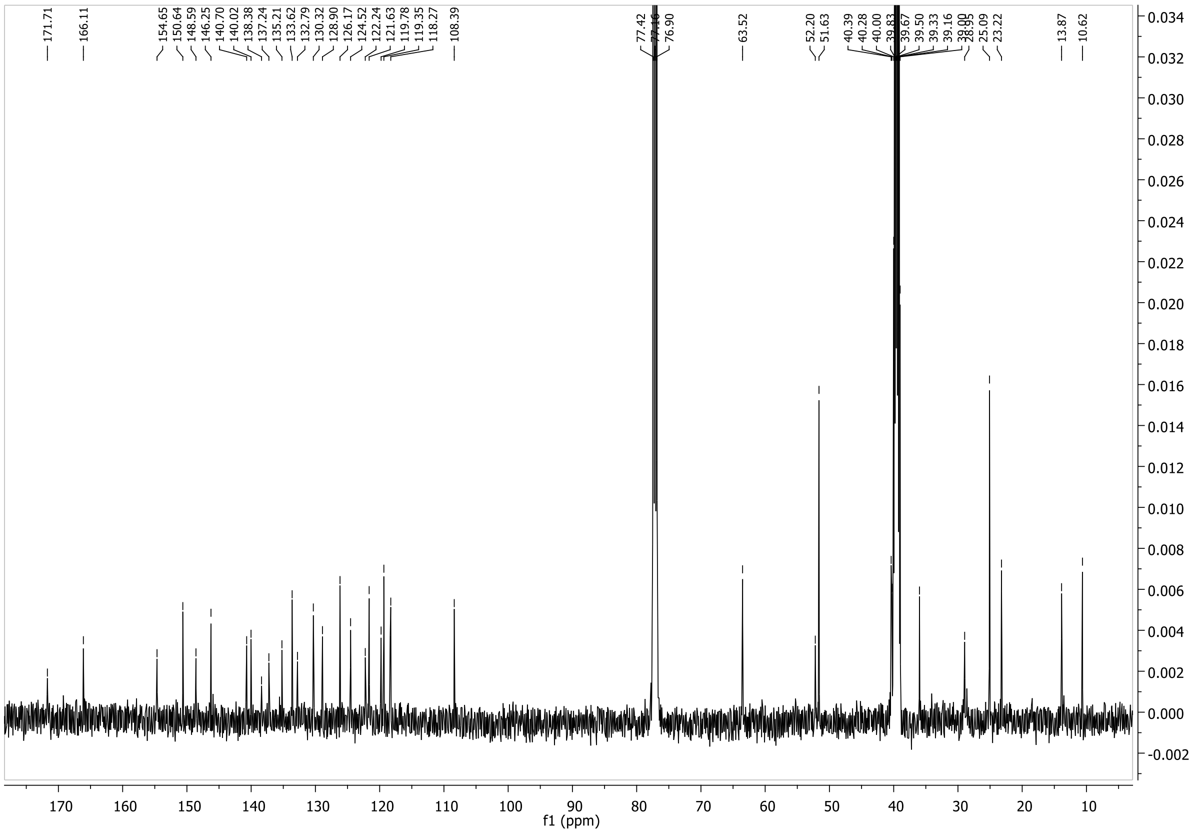

Compound 15 (DMSO-d6)

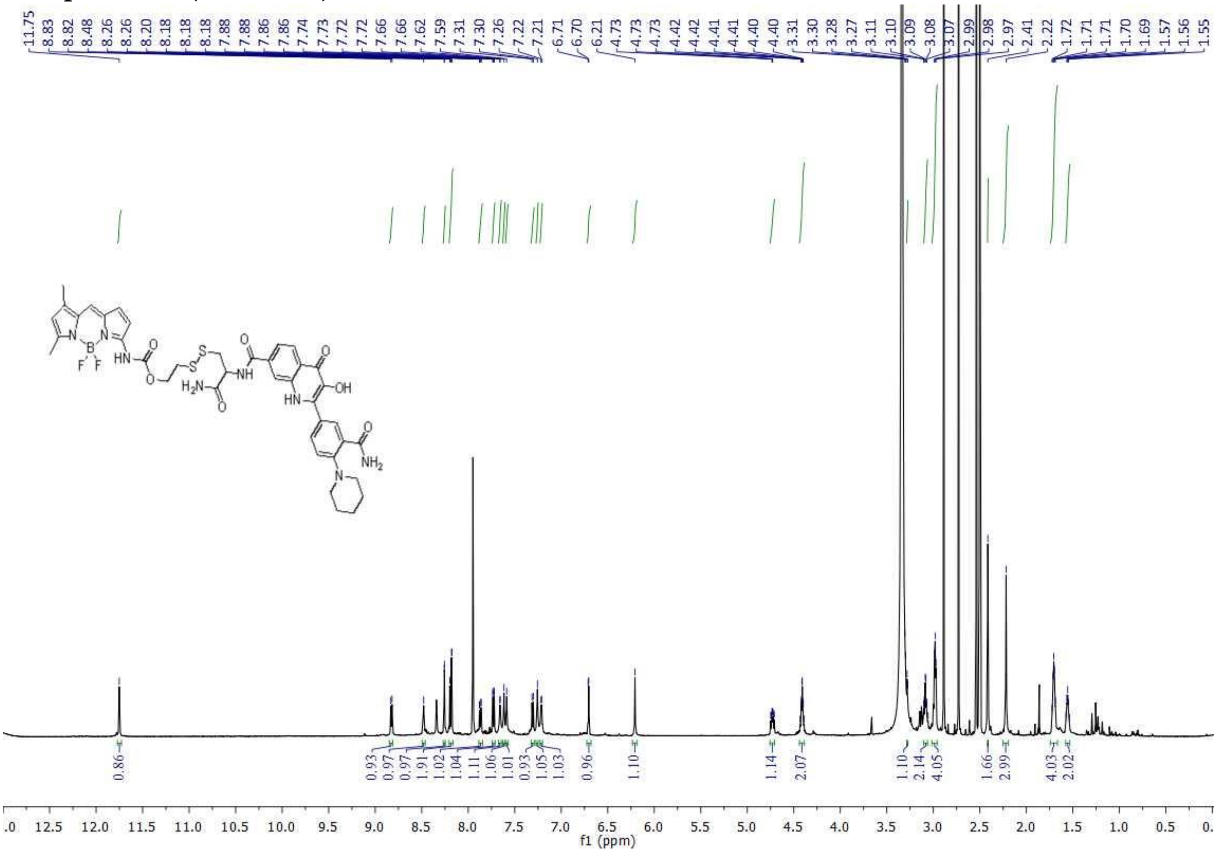

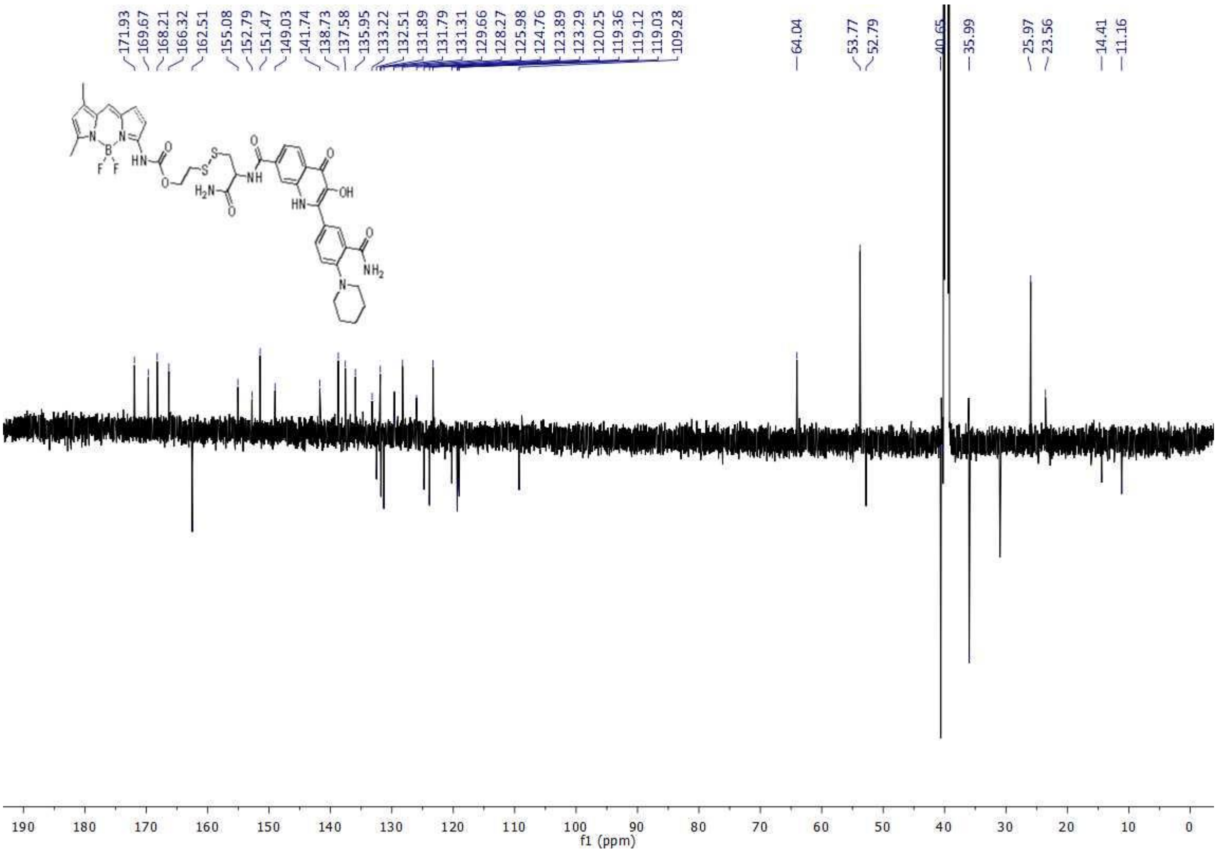

Compound 18 ($CDCl_3$)

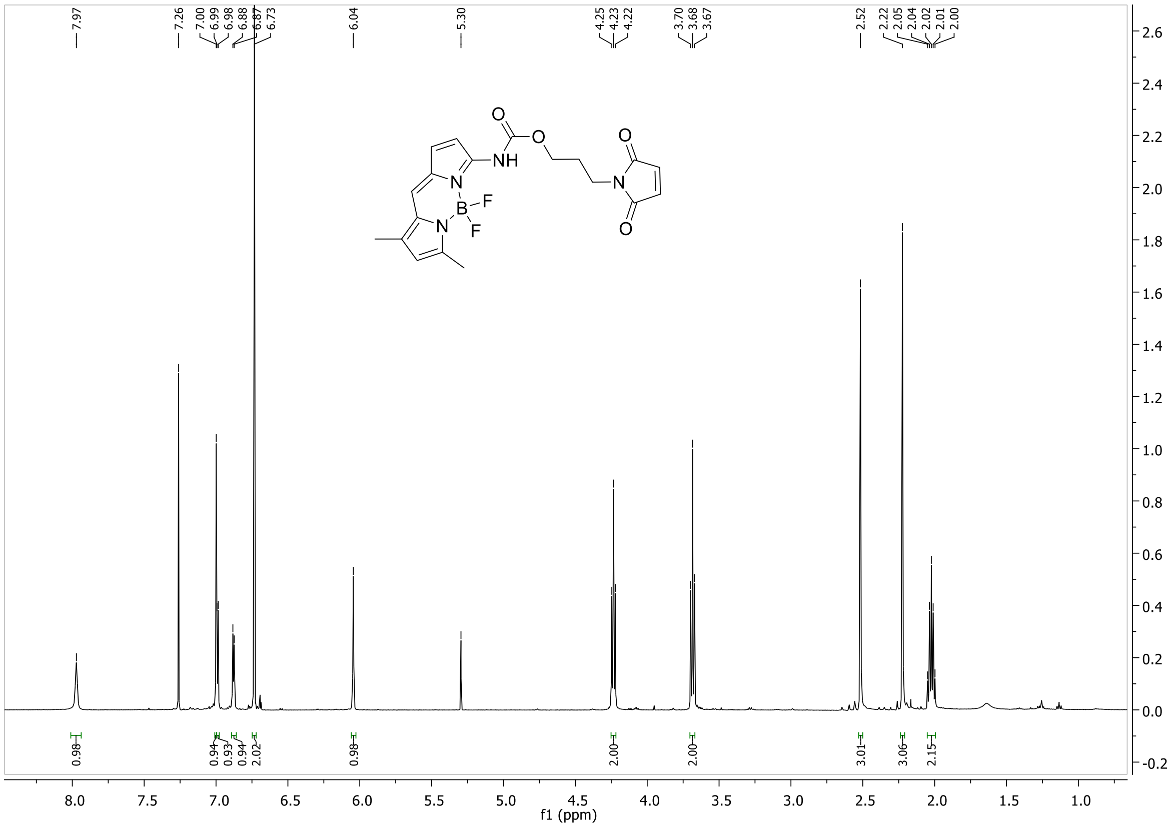

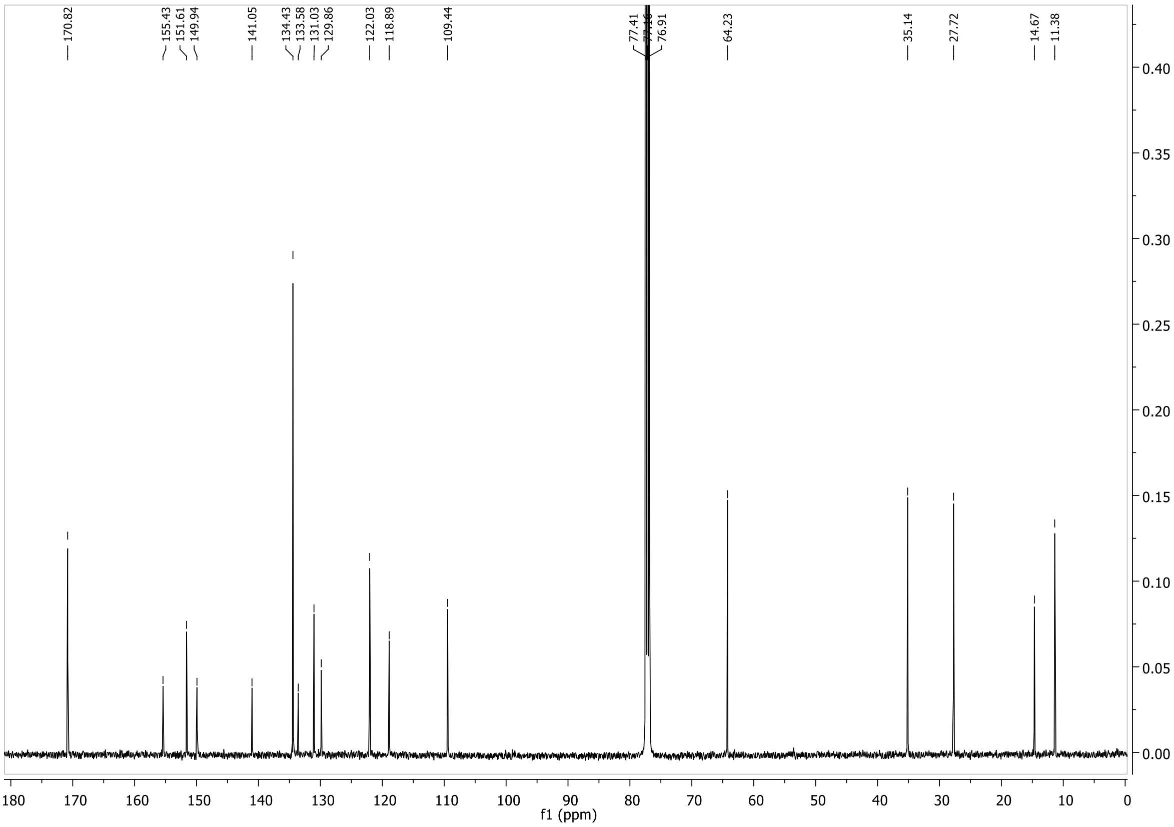

Supplement: Supplementary file 1 — Supporting Information [file OPEN-10-1104-s001.pdf]
